# Supplementary figures and images for: eIF3 engages with 3’-UTR termini of highly translated mRNAs
Source: eLife. 2025 Jan 29;13:RP102977. doi: 10.7554/eLife.102977 (PMC11778930; doi:10.7554/eLife.102977)

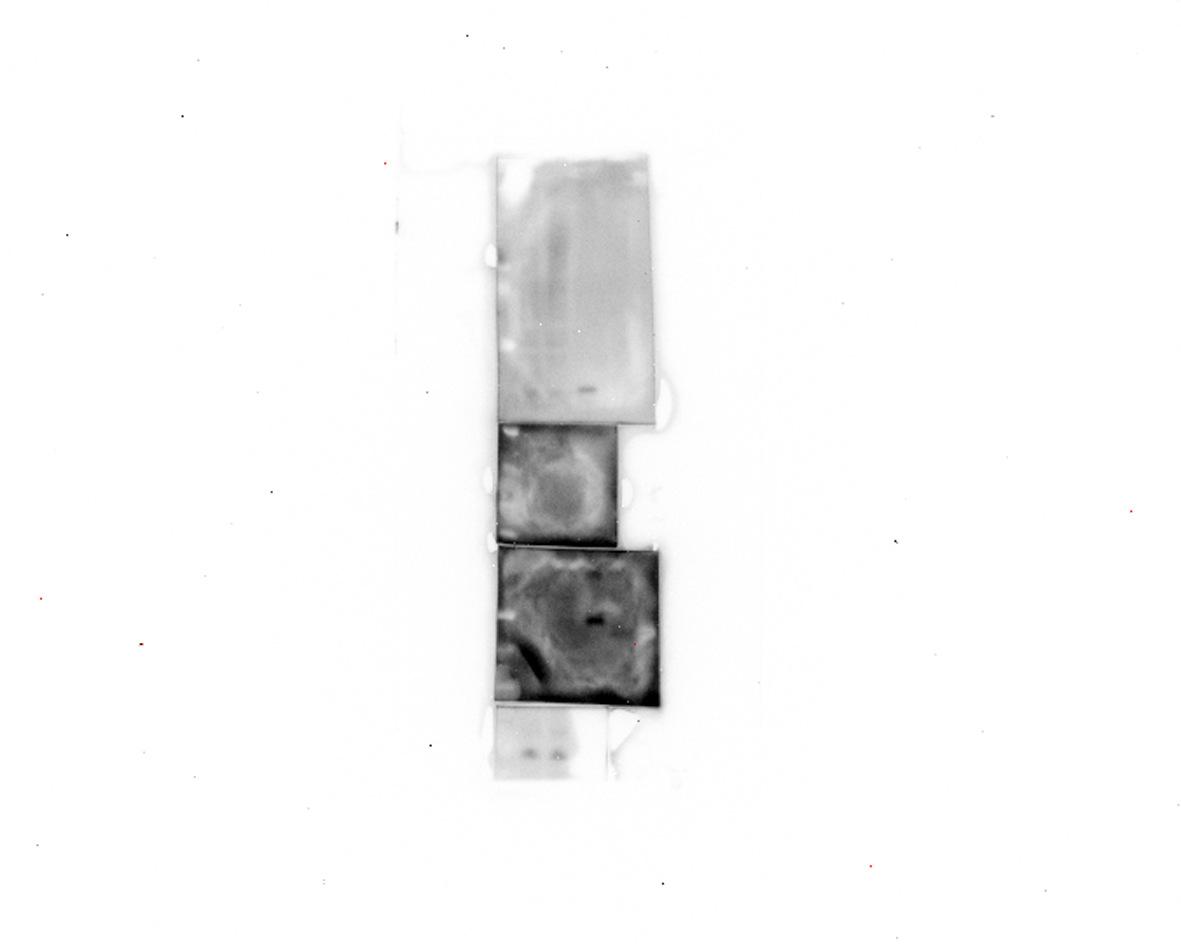

Supplement: Figure 1—source data 1. [file elife-102977-fig1-data1.zip › Figure1-source data 1/Fig1G_EIF3I_original.tif]

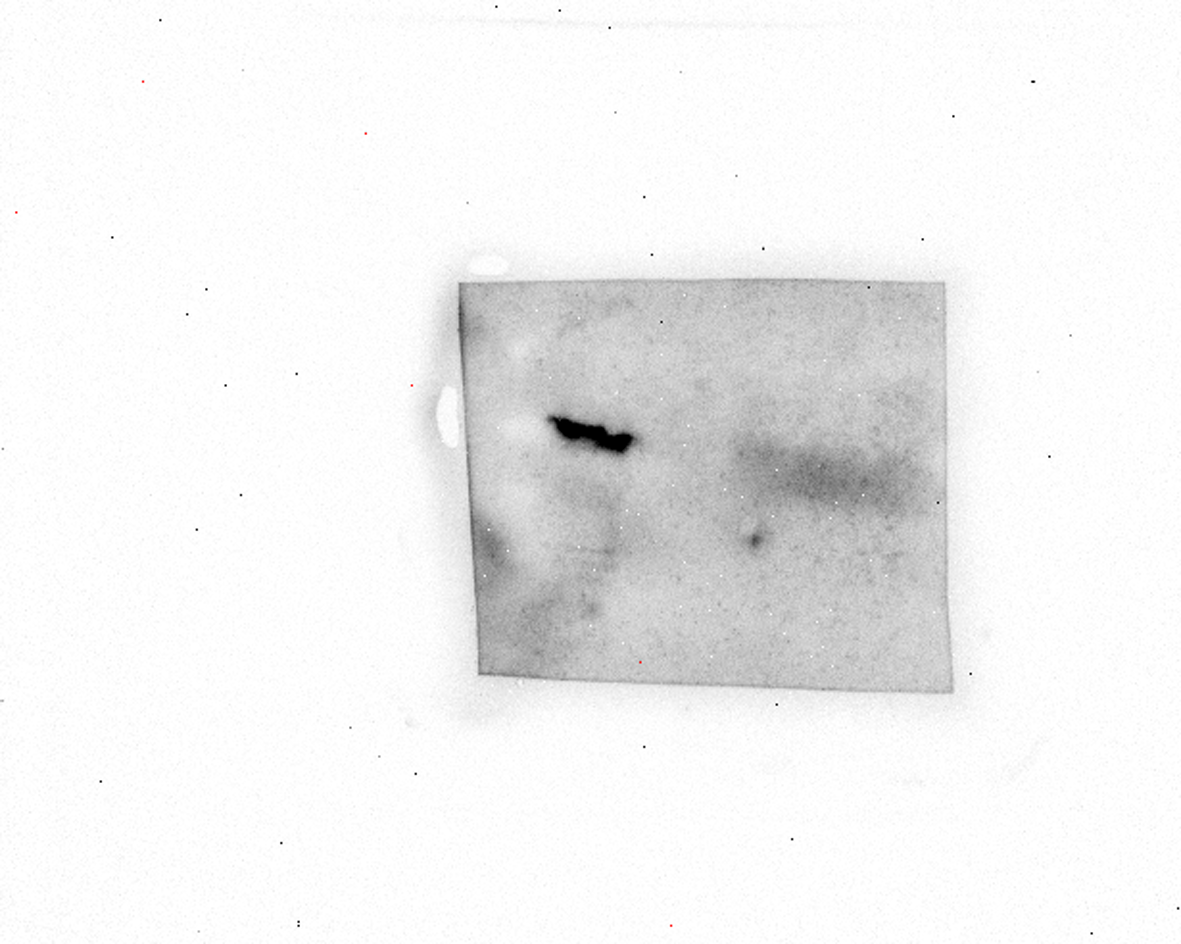

Supplement: Figure 1—source data 1. [file elife-102977-fig1-data1.zip › Figure1-source data 1/Fig1C_Oct4_original.tif]

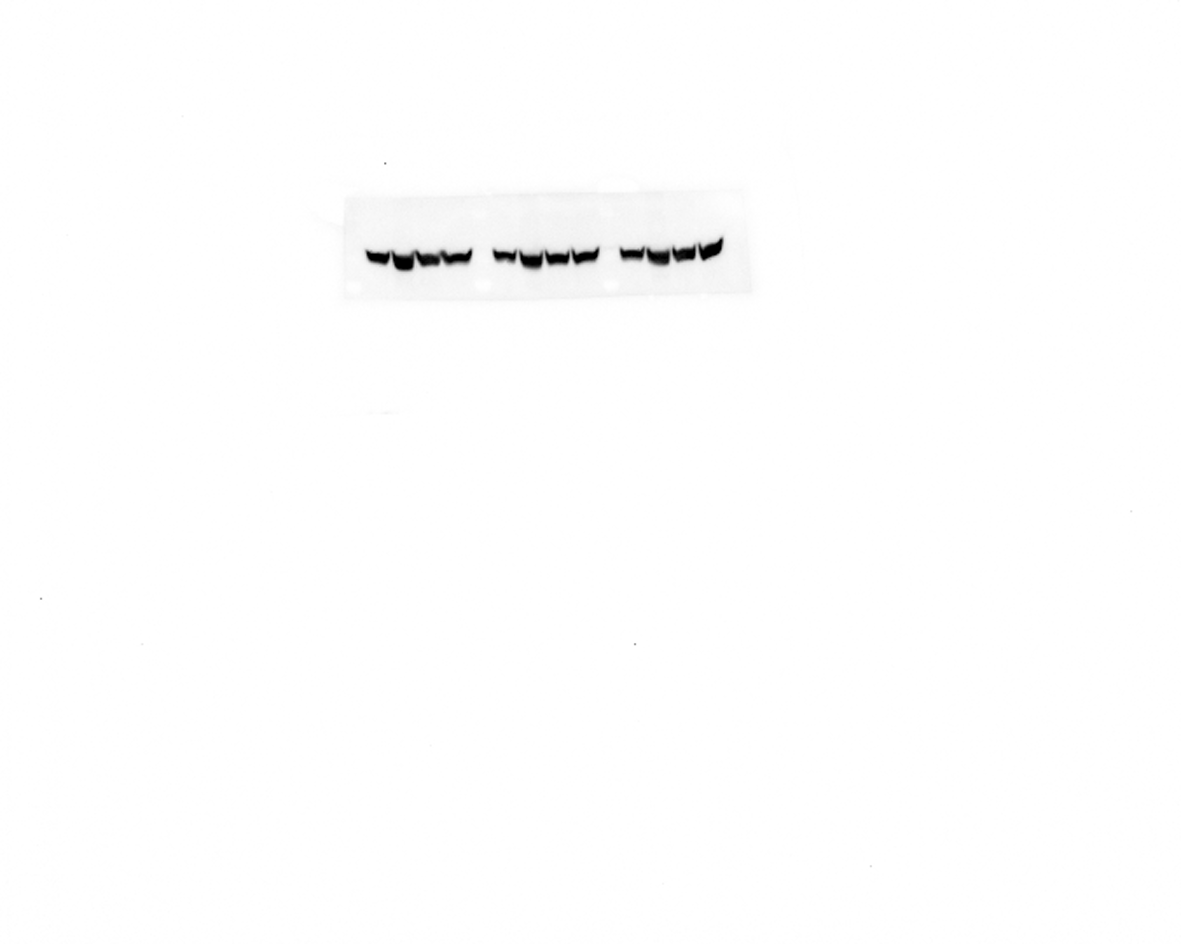

Supplement: Figure 1—source data 1. [file elife-102977-fig1-data1.zip › Figure1-source data 1/Fig1C_Hsp90_original.tif]

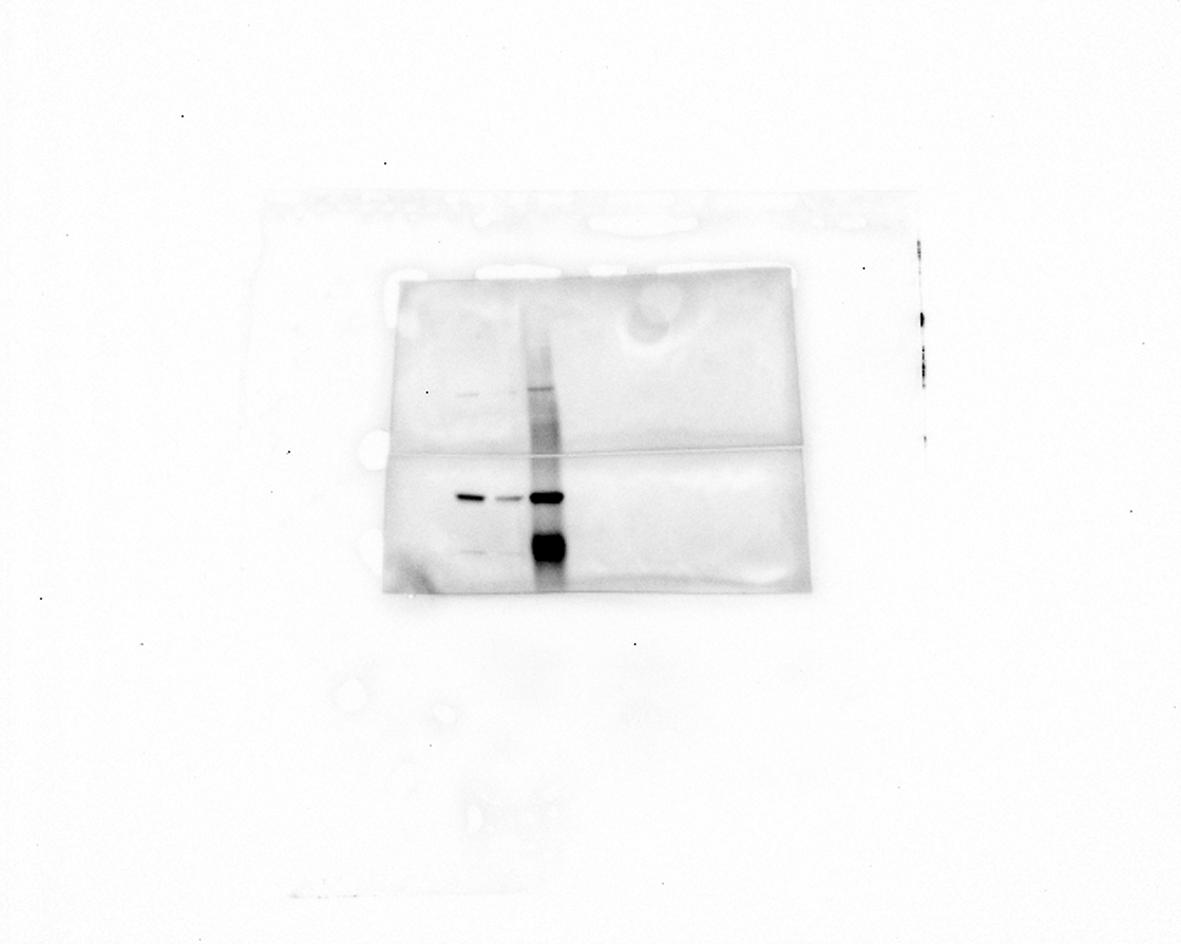

Supplement: Figure 1—source data 1. [file elife-102977-fig1-data1.zip › Figure1-source data 1/Fig1G_EIF3C_EIF3L_original.tif]

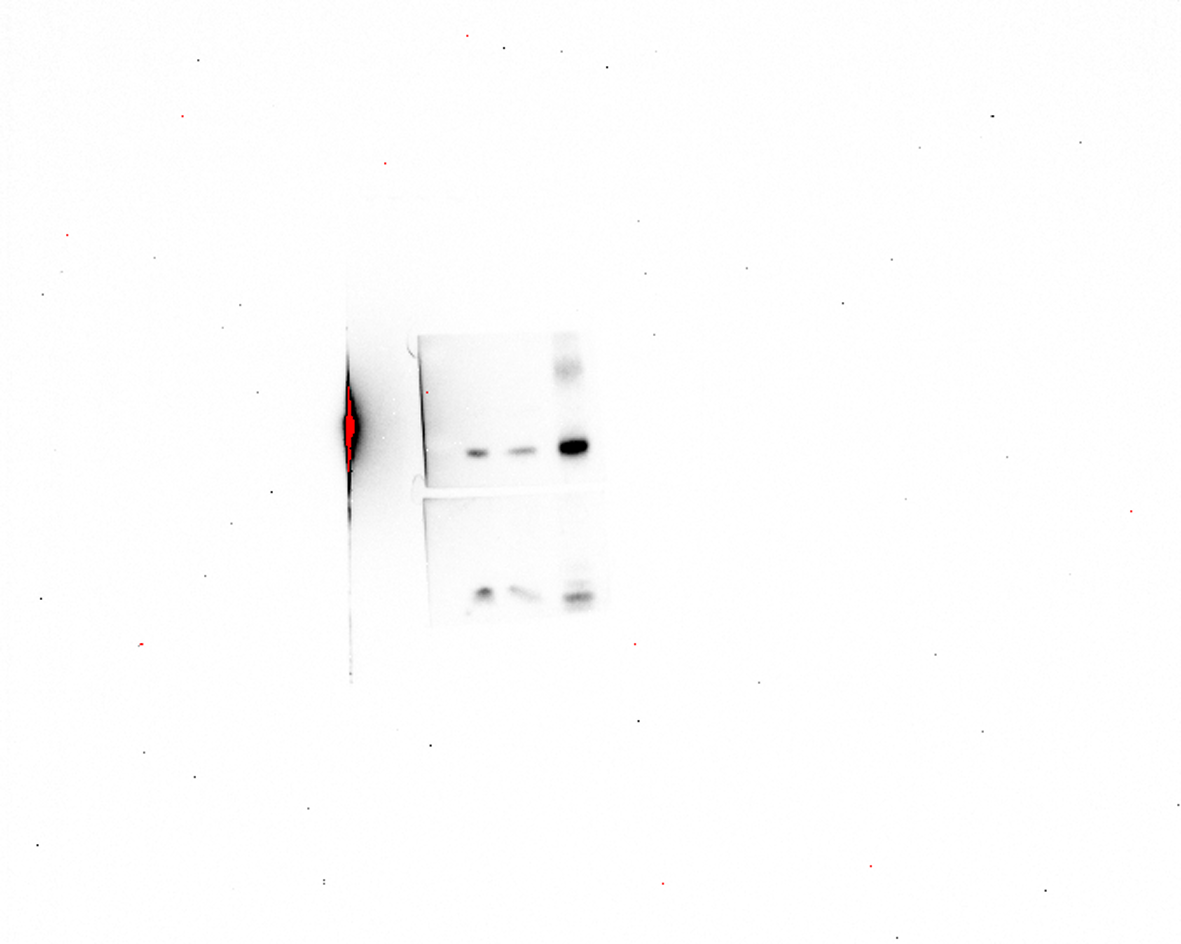

Supplement: Figure 1—source data 1. [file elife-102977-fig1-data1.zip › Figure1-source data 1/Fig1G_EIF3E_original.tif]

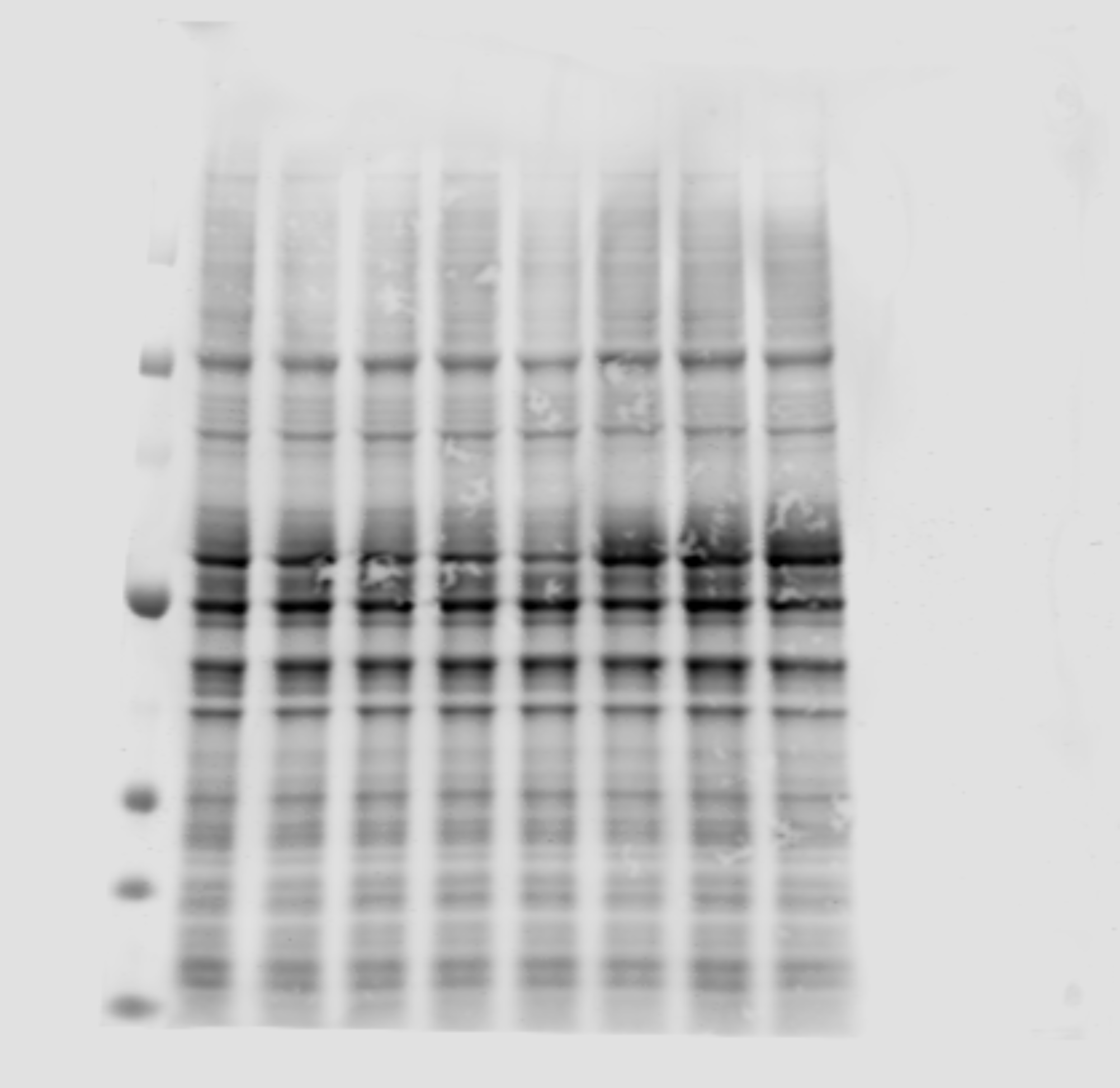

Supplement: Figure 1—source data 1. [file elife-102977-fig1-data1.zip › Figure1-source data 1/Fig1D_totalproteinstain_original.tif]

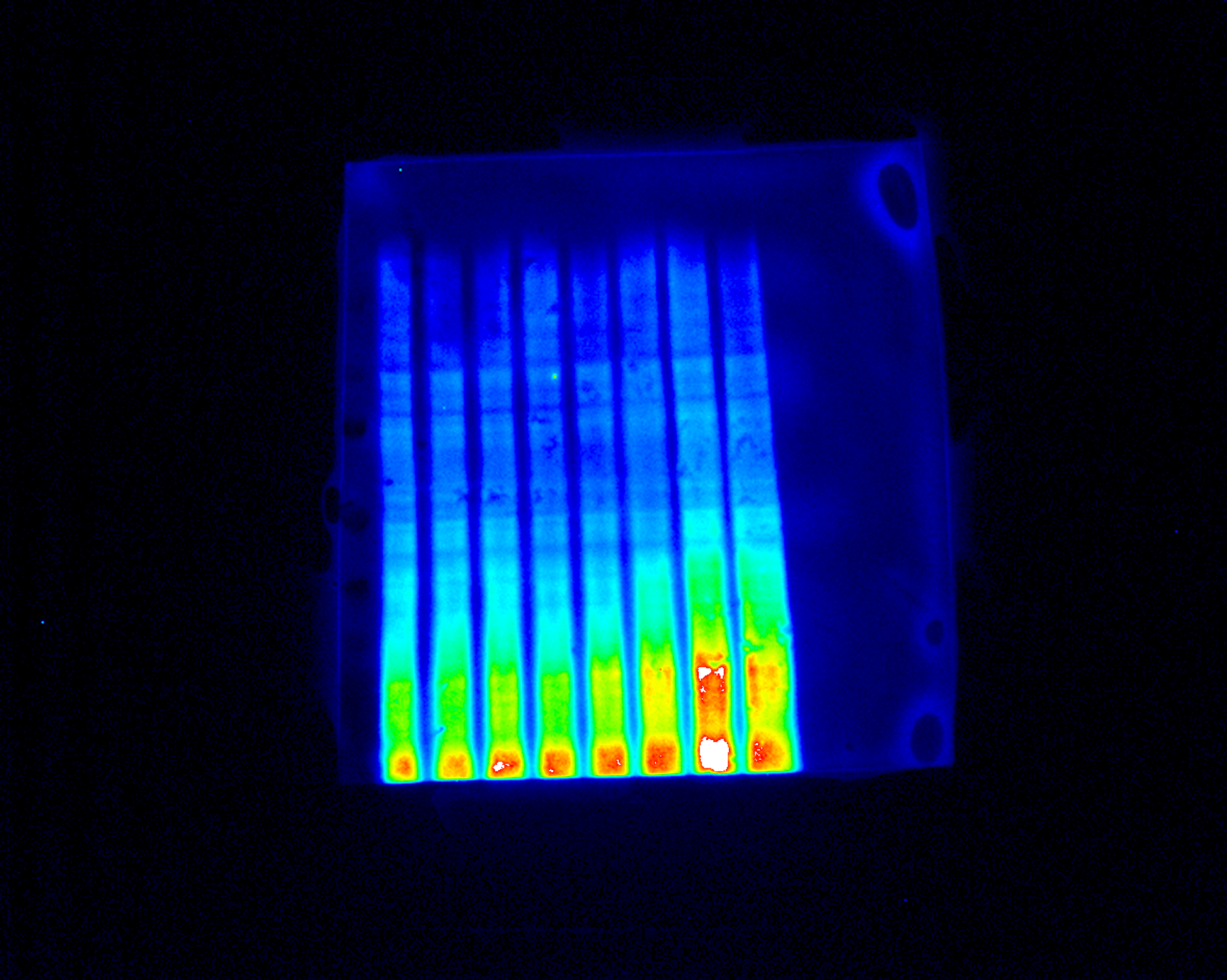

Supplement: Figure 1—source data 1. [file elife-102977-fig1-data1.zip › Figure1-source data 1/Fig1D_puromycin_original.tif]

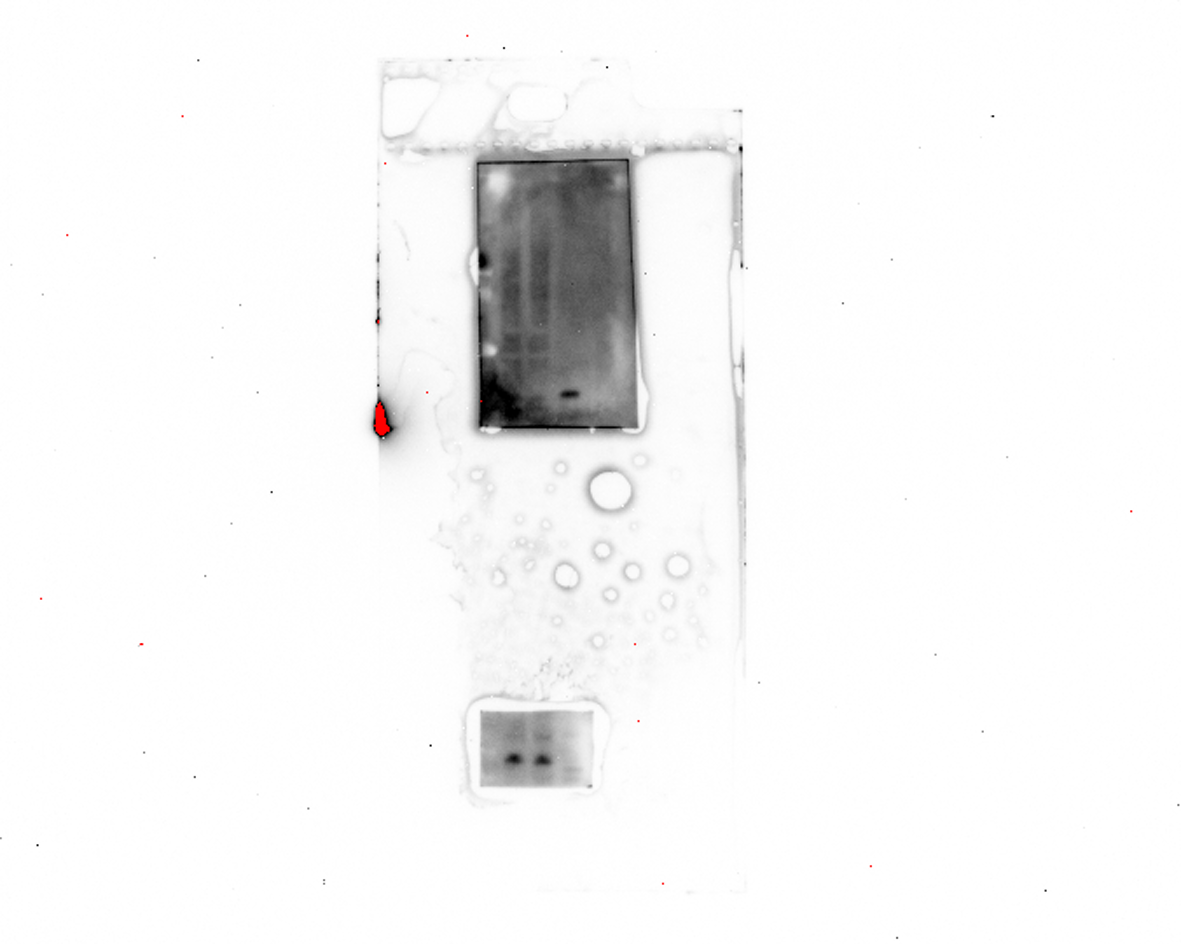

Supplement: Figure 1—source data 1. [file elife-102977-fig1-data1.zip › Figure1-source data 1/Fig1G_EIF4E_original.tif]

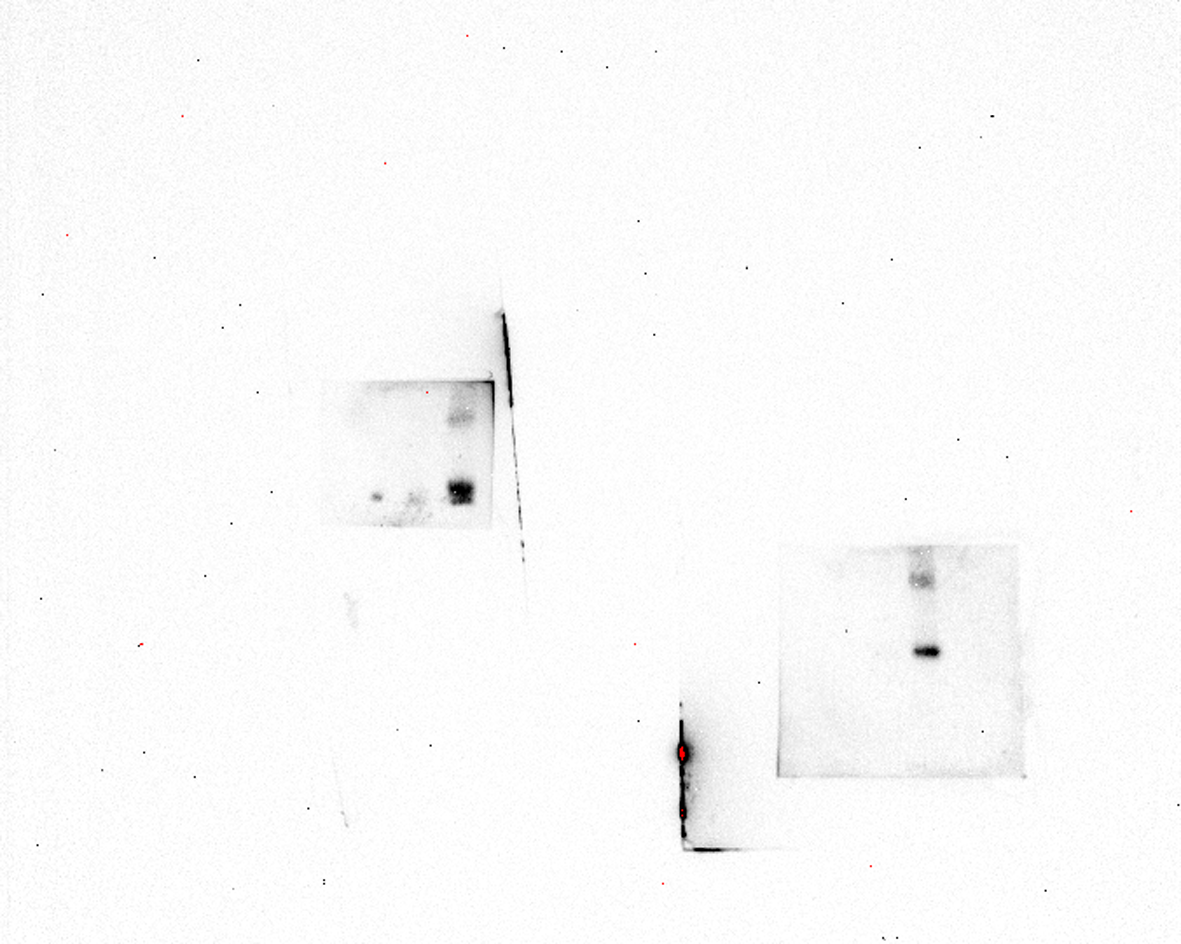

Supplement: Figure 1—source data 1. [file elife-102977-fig1-data1.zip › Figure1-source data 1/Fig1G_EIF3F_original.tif]

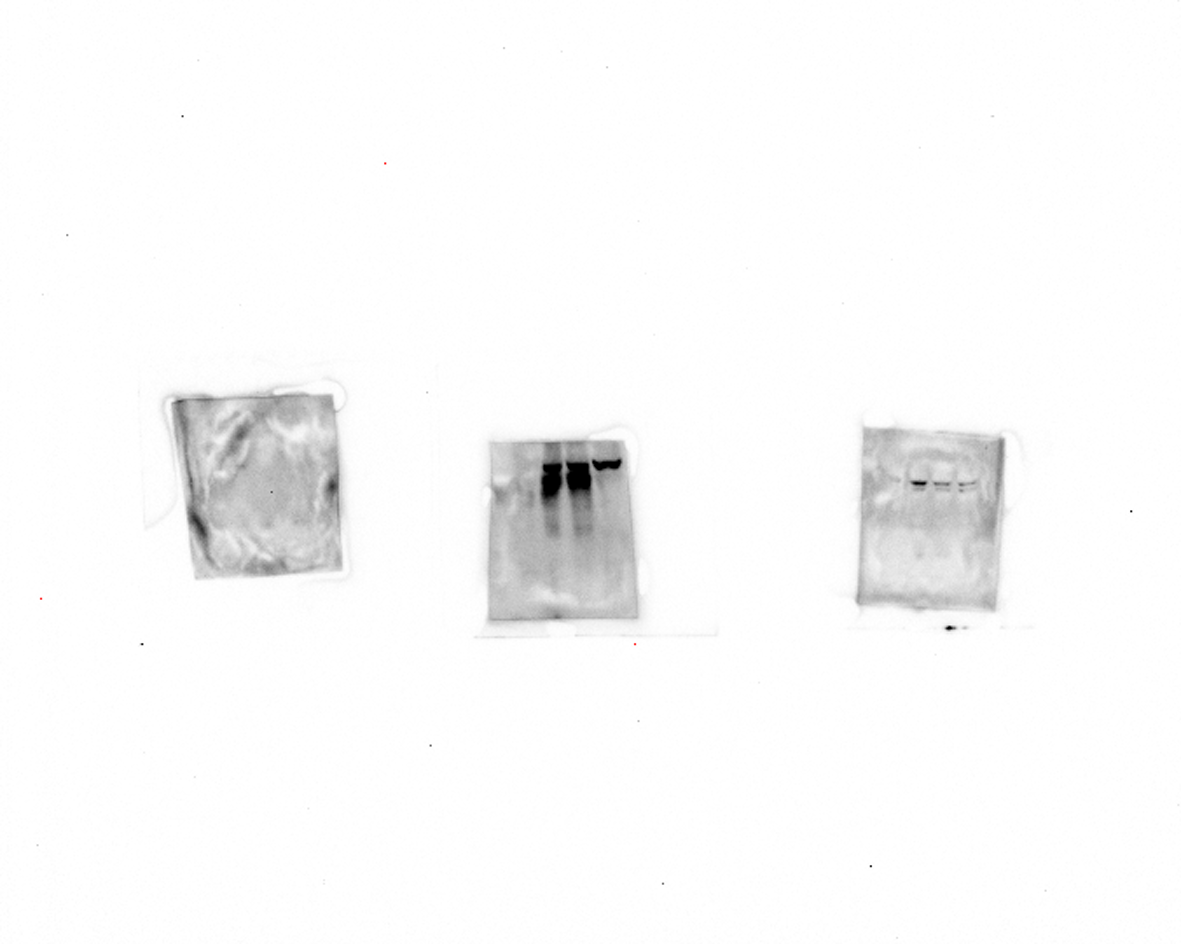

Supplement: Figure 1—source data 1. [file elife-102977-fig1-data1.zip › Figure1-source data 1/Fig1C_Pax6_Sox1_original.tif]

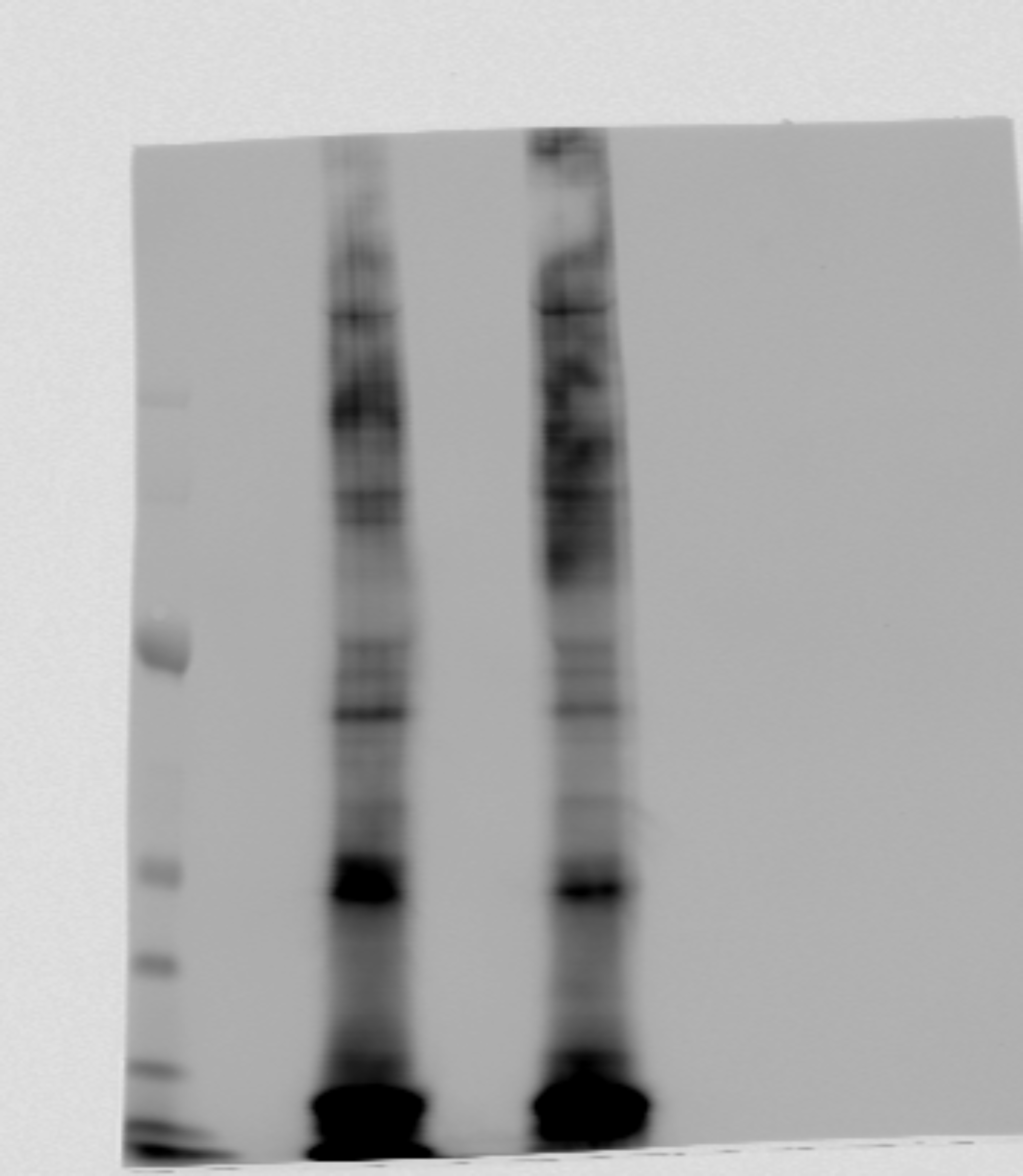

Supplement: Figure 1—source data 1. [file elife-102977-fig1-data1.zip › Figure1-source data 1/Fig1H_original.tif]

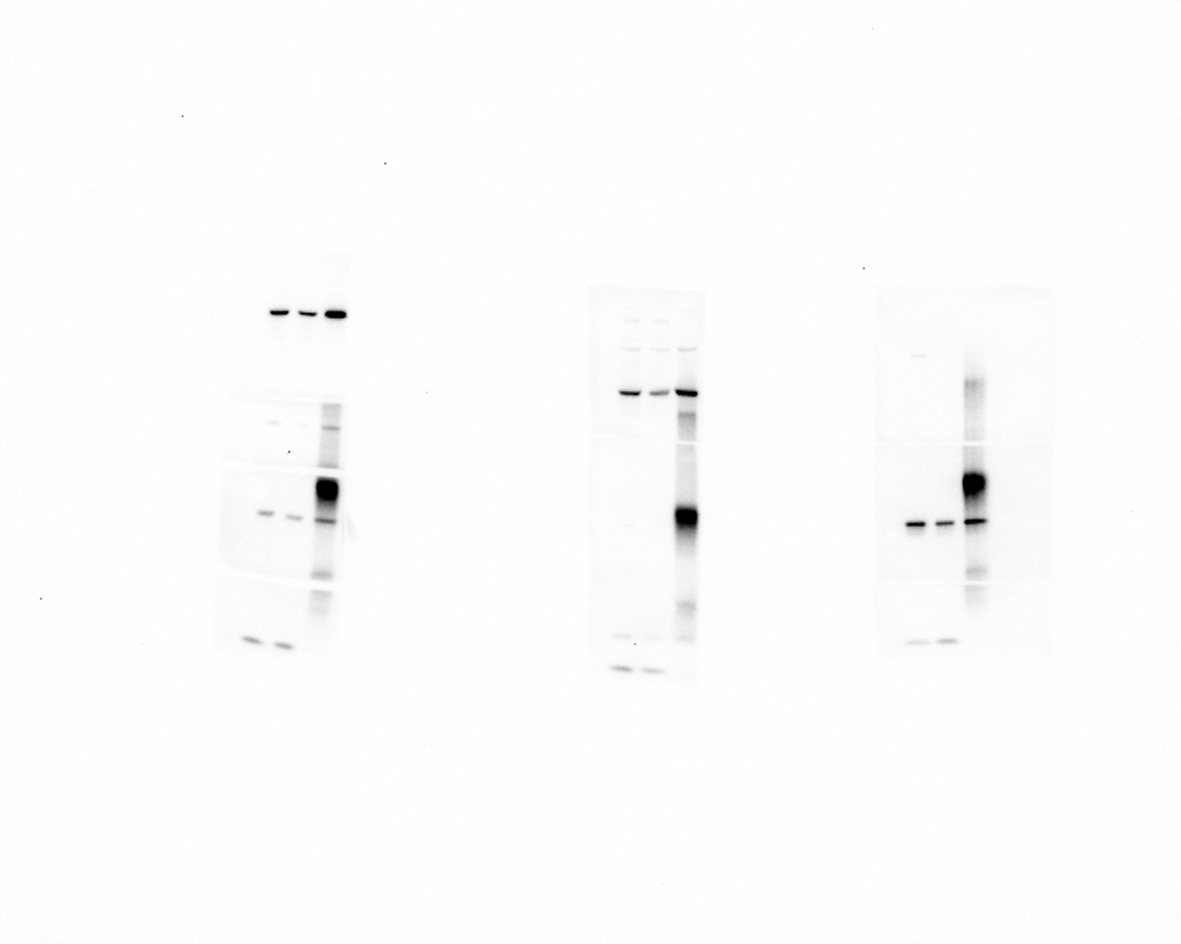

Supplement: Figure 1—source data 1. [file elife-102977-fig1-data1.zip › Figure1-source data 1/Fig1G_EIF3A_EIF3B_EIF3D_EIF3H_EIF3K_EIF3M_RPS19_original.tif]

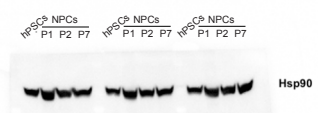

Supplement: Figure 1—source data 2. [file elife-102977-fig1-data2.zip › Figure1-source data 2/Fig1C_Hsp90_labeled.pdf]

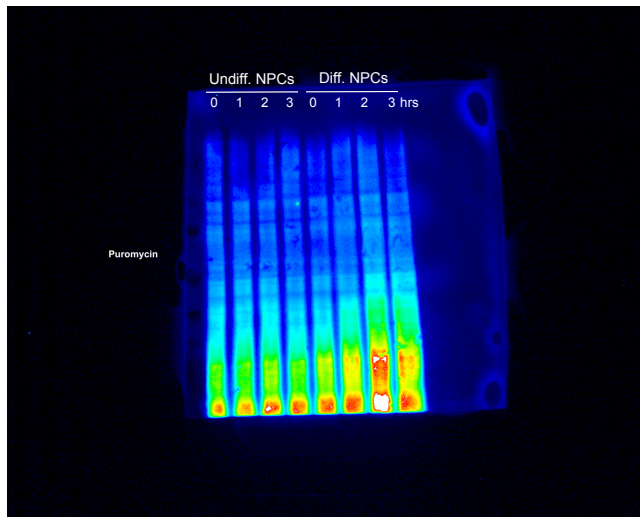

Supplement: Figure 1—source data 2. [file elife-102977-fig1-data2.zip › Figure1-source data 2/Fig1D_puromycin_labeled.pdf]

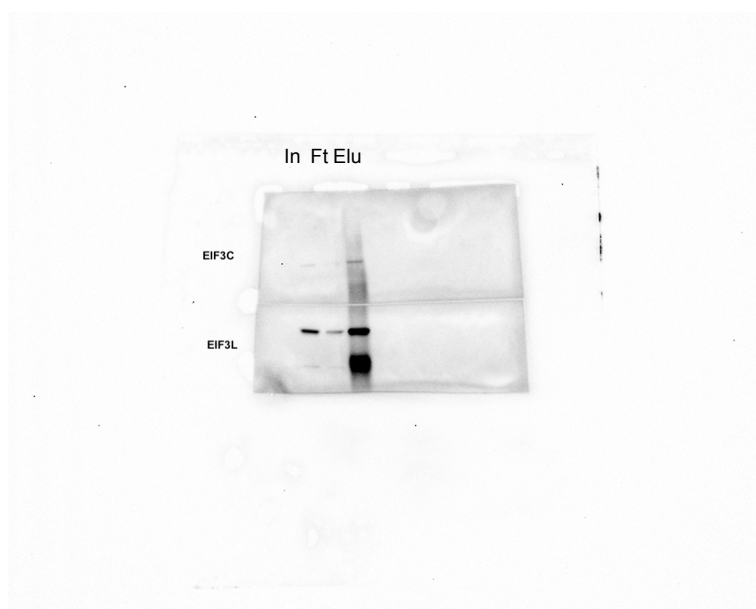

Supplement: Figure 1—source data 2. [file elife-102977-fig1-data2.zip › Figure1-source data 2/Fig1G_EIF3C_EIF3L_labeled.pdf]

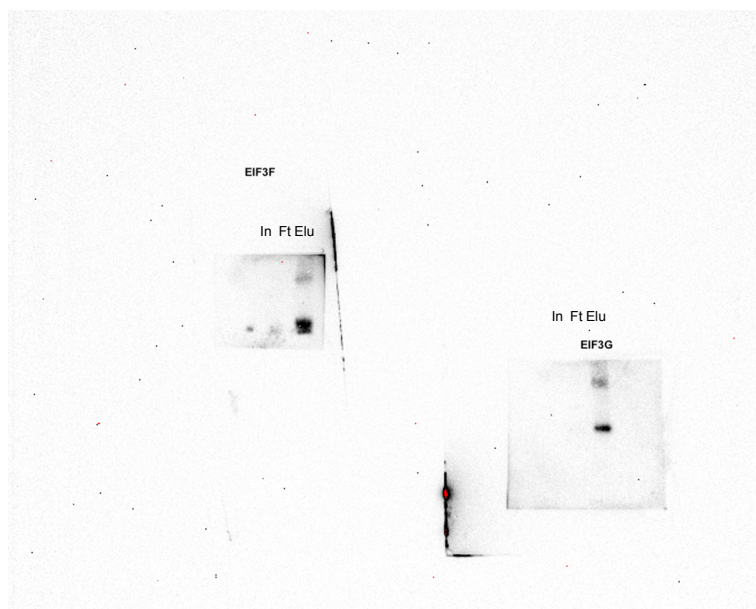

Supplement: Figure 1—source data 2. [file elife-102977-fig1-data2.zip › Figure1-source data 2/Fig1G_EIF3F_labeled.pdf]

hPSCs NPCs  
P1 P2 P7

Oct4

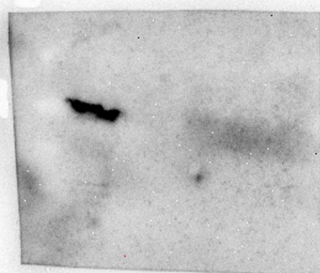

Supplement: Figure 1—source data 2. [file elife-102977-fig1-data2.zip › Figure1-source data 2/Fig1C_Oct4_labeled.pdf]

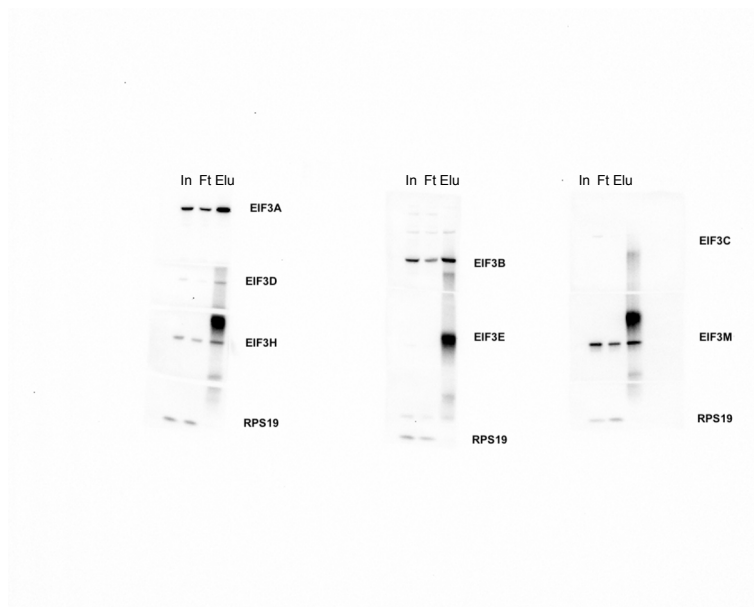

Supplement: Figure 1—source data 2. [file elife-102977-fig1-data2.zip › Figure1-source data 2/Fig1G_EIF3A_EIF3B_EIF3D_EIF3H_EIF3K_EIF3M_RPS19_labeled.pdf]

In FtElu

EIF3I

EIF3J

EIF3G

EIF4E

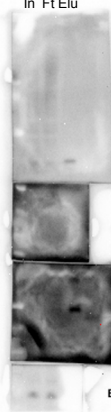

Supplement: Figure 1—source data 2. [file elife-102977-fig1-data2.zip › Figure1-source data 2/Fig1G_EIF3I_labeled.pdf]

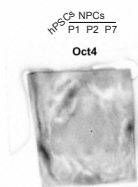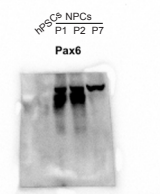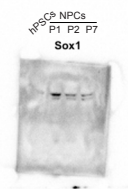

Supplement: Figure 1—source data 2. [file elife-102977-fig1-data2.zip › Figure1-source data 2/Fig1C_Pax6_Sox1_labeled.pdf]

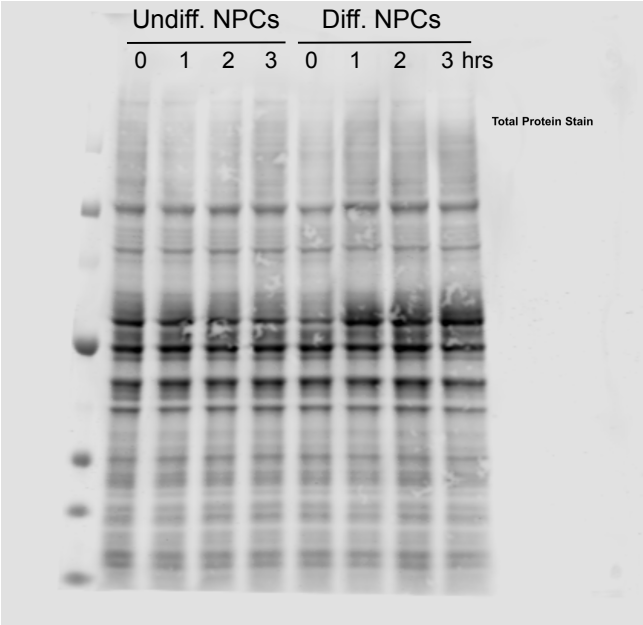

Supplement: Figure 1—source data 2. [file elife-102977-fig1-data2.zip › Figure1-source data 2/Fig1D_totalproteinstain_labeled.pdf]

# NPCs

Diff.

Undiff.

260

140

100

70

50

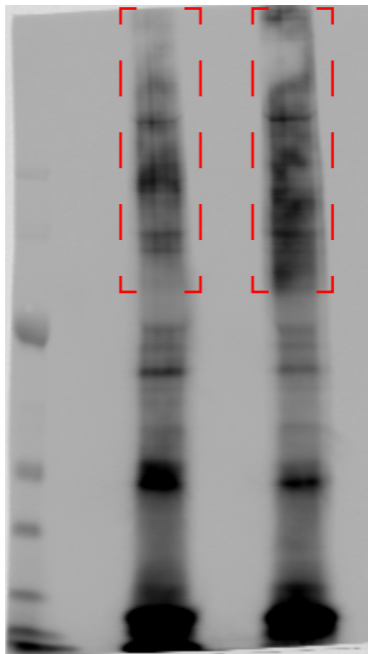

Supplement: Figure 1—source data 2. [file elife-102977-fig1-data2.zip › Figure1-source data 2/Fig1H_labeled.pdf]

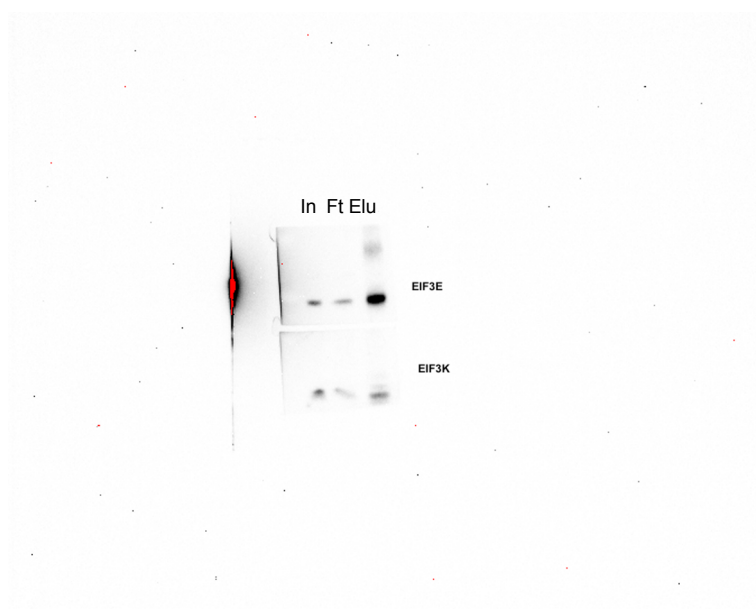

Supplement: Figure 1—source data 2. [file elife-102977-fig1-data2.zip › Figure1-source data 2/Fig1G_EIF3E_labeled.pdf]

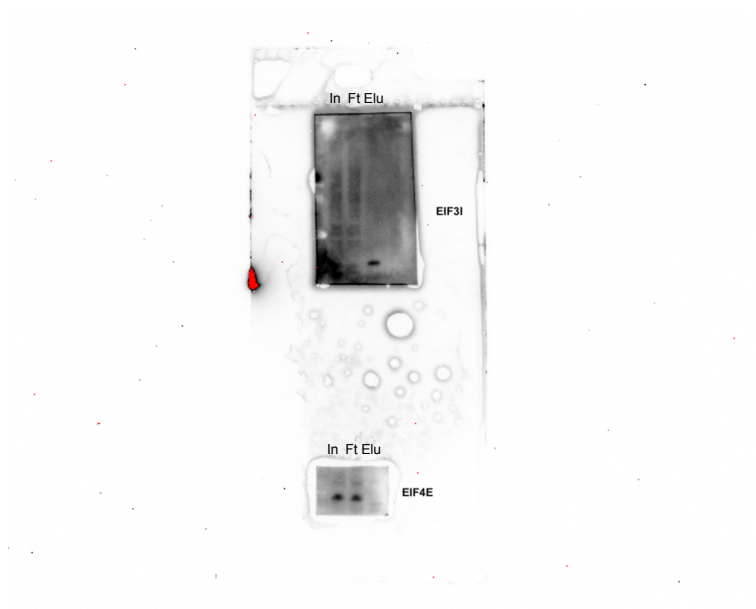

Supplement: Figure 1—source data 2. [file elife-102977-fig1-data2.zip › Figure1-source data 2/Fig1G_EIF4E_labeled.pdf]

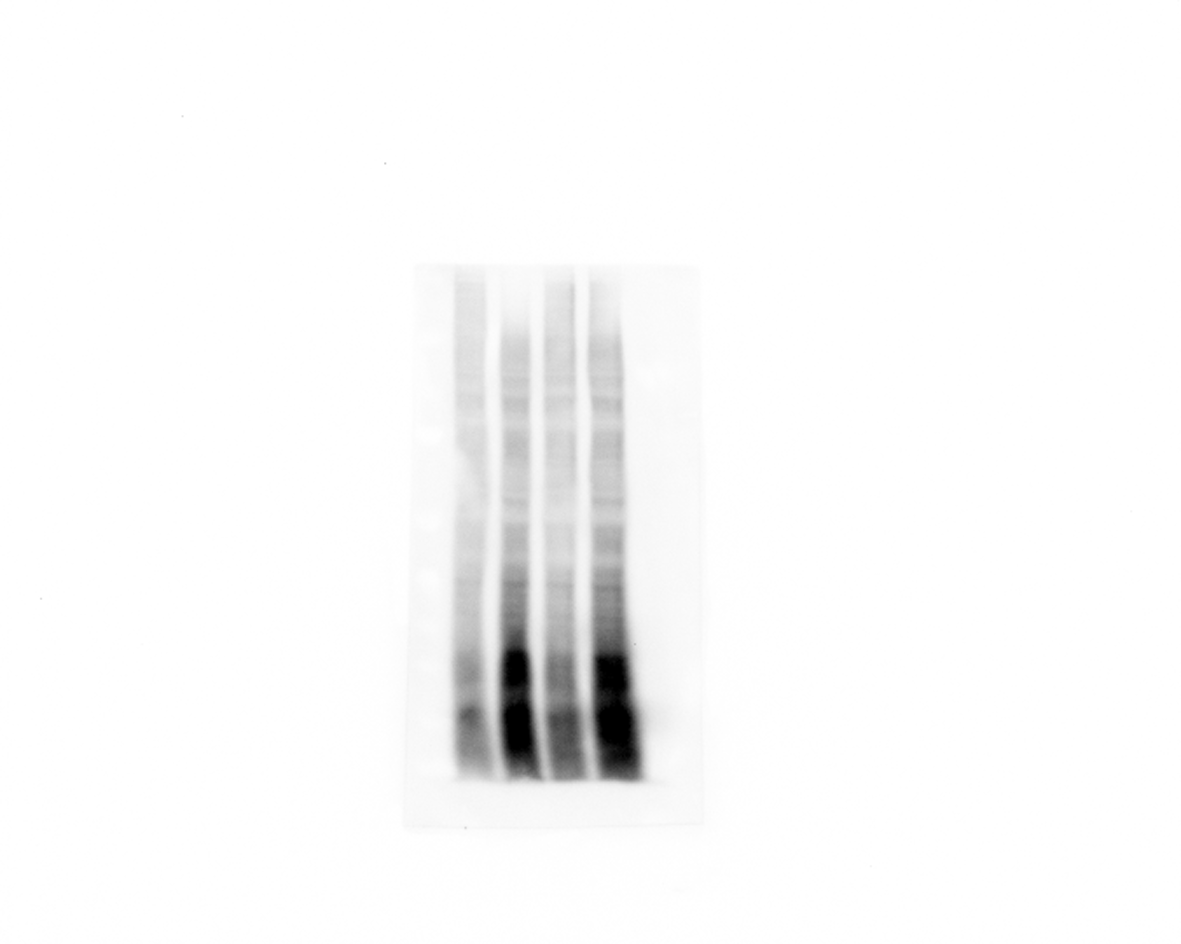

Supplement: Figure 1—figure supplement 1—source data 1. [file elife-102977-fig1-figsupp1-data1.zip › Figure1-figure supplement 1-source data 1/Fig1Supp1B_Puromycin_original.tif]

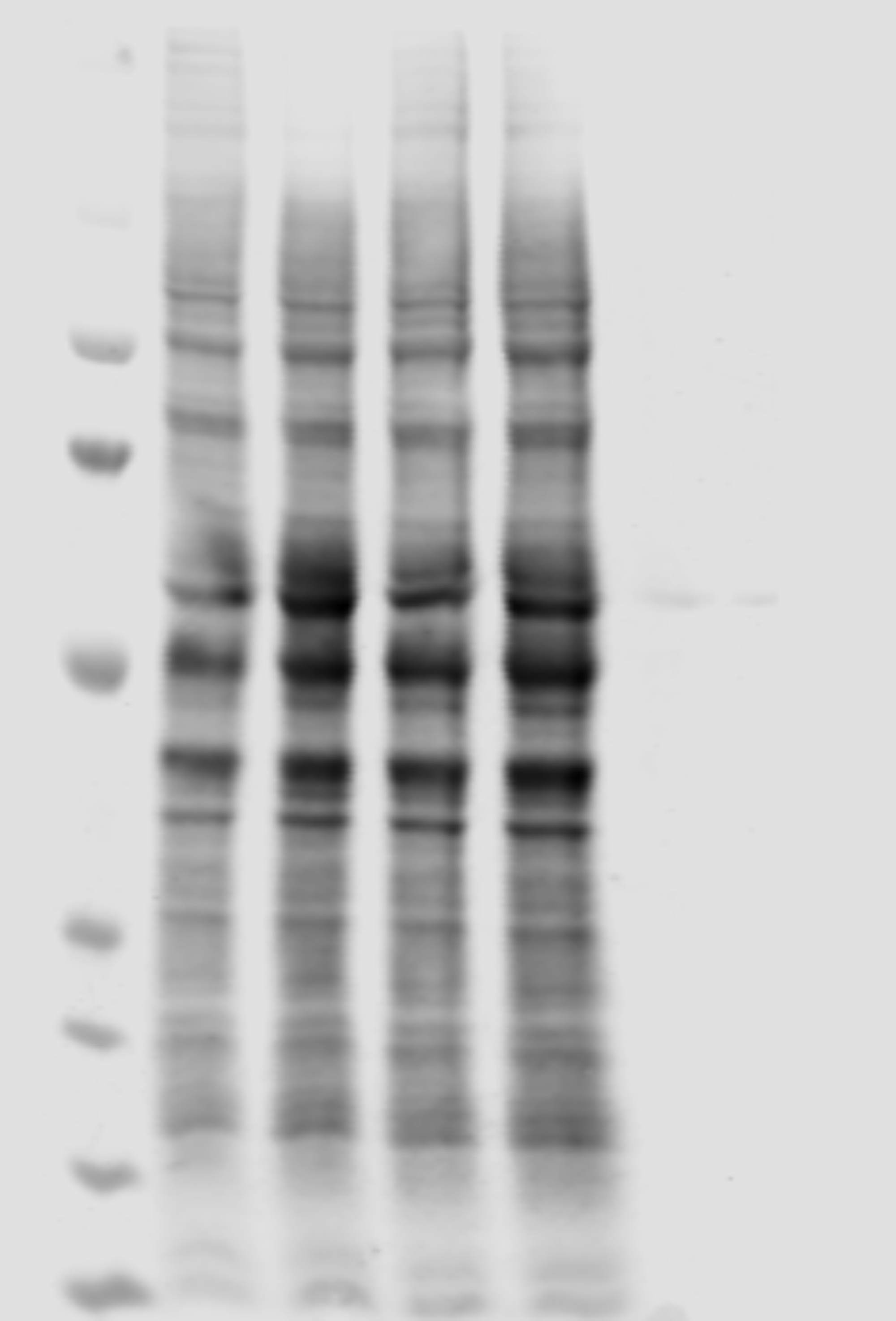

Supplement: Figure 1—figure supplement 1—source data 1. [file elife-102977-fig1-figsupp1-data1.zip › Figure1-figure supplement 1-source data 1/Fig1Supp1B_Totalproteinstain_original.tif]

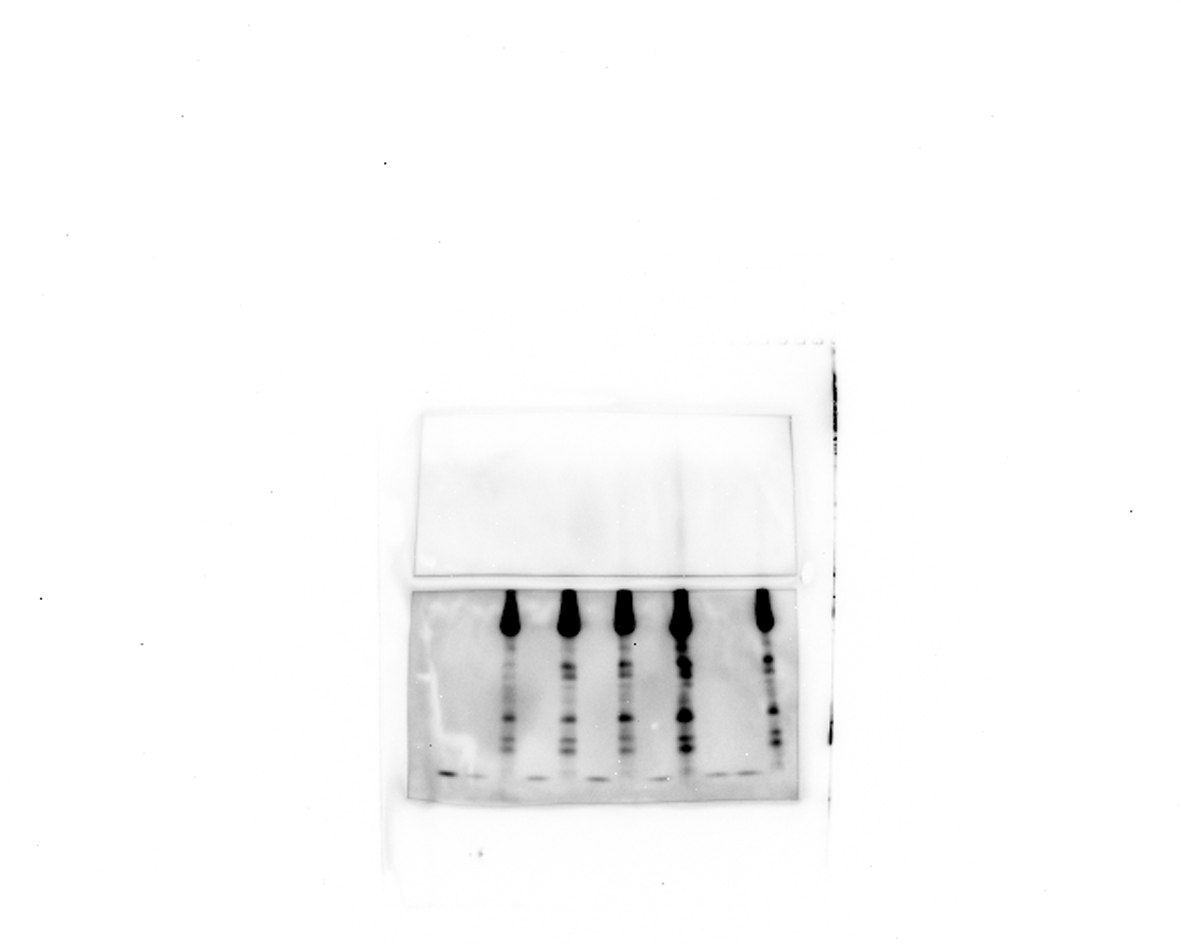

Supplement: Figure 1—figure supplement 5—source data 1. [file elife-102977-fig1-figsupp5-data1.zip › Figure1-figure supplement 5-source data 1/Fig1Supp5_RPS19_original.tif]

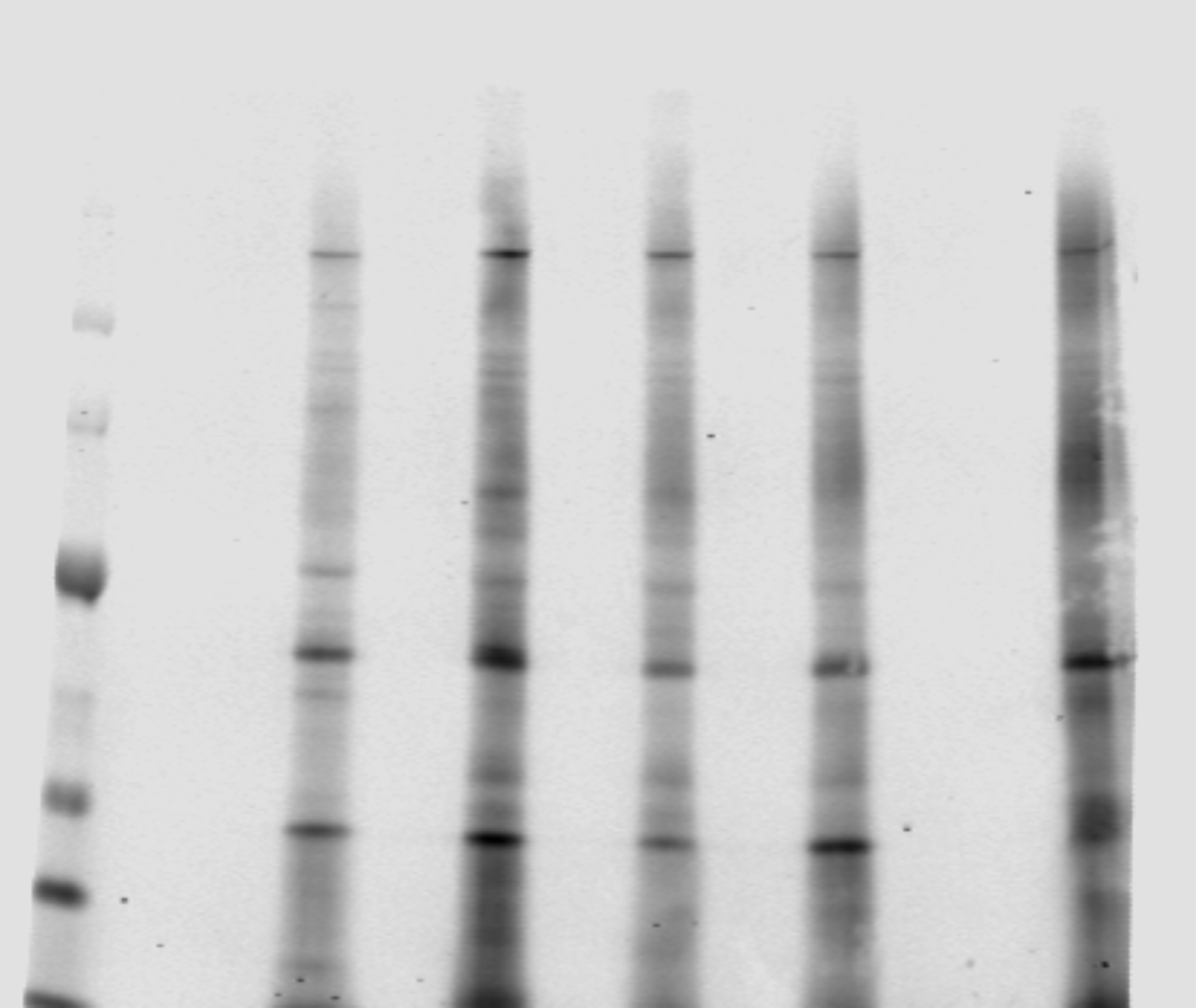

Supplement: Figure 1—figure supplement 5—source data 1. [file elife-102977-fig1-figsupp5-data1.zip › Figure1-figure supplement 5-source data 1/Fig1Supp5_IR-RNAs_original.tif]

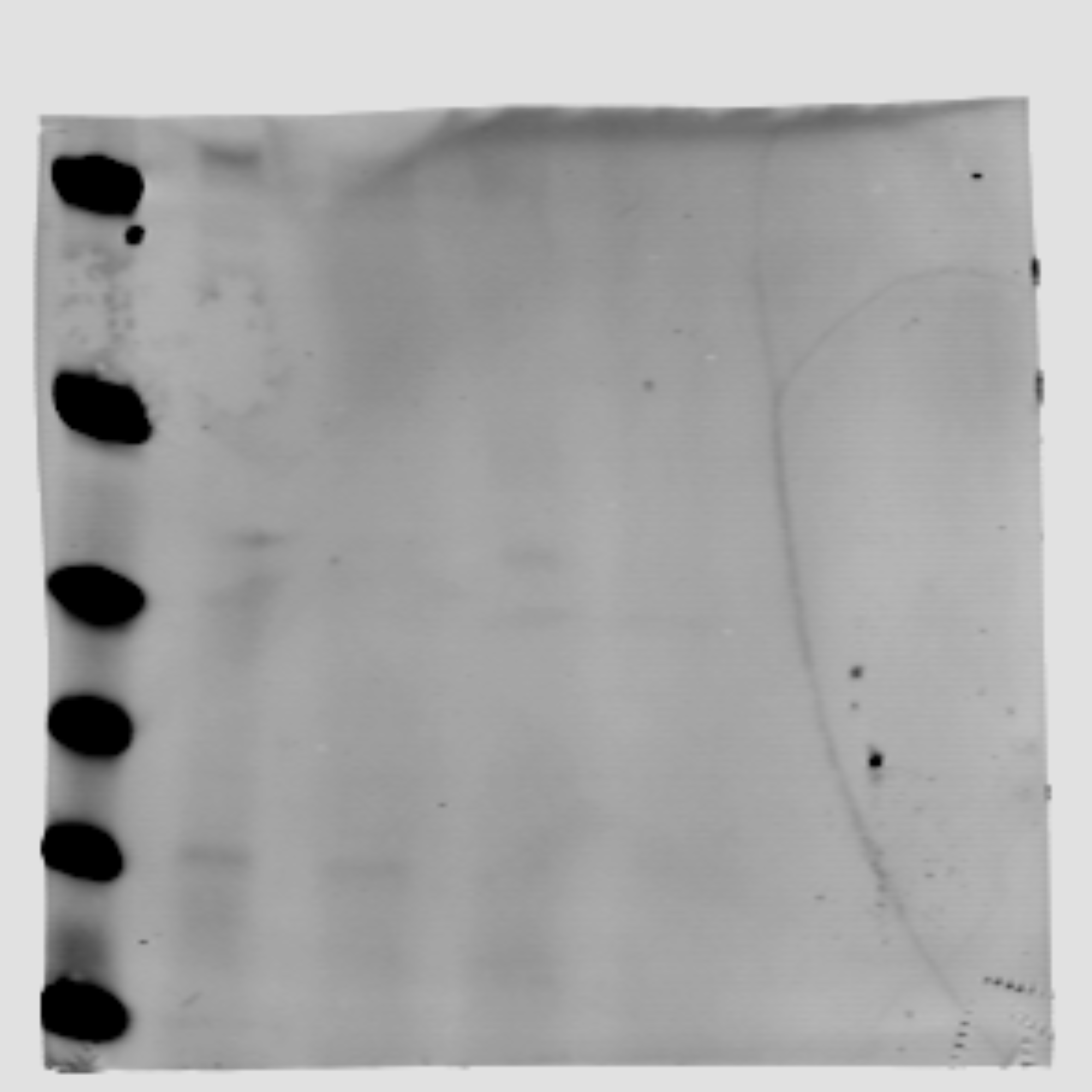

Supplement: Figure 3—source data 1. [file elife-102977-fig3-data1.zip › Figure3-source data 1/Fig3D_SLC38A2_original.tif]

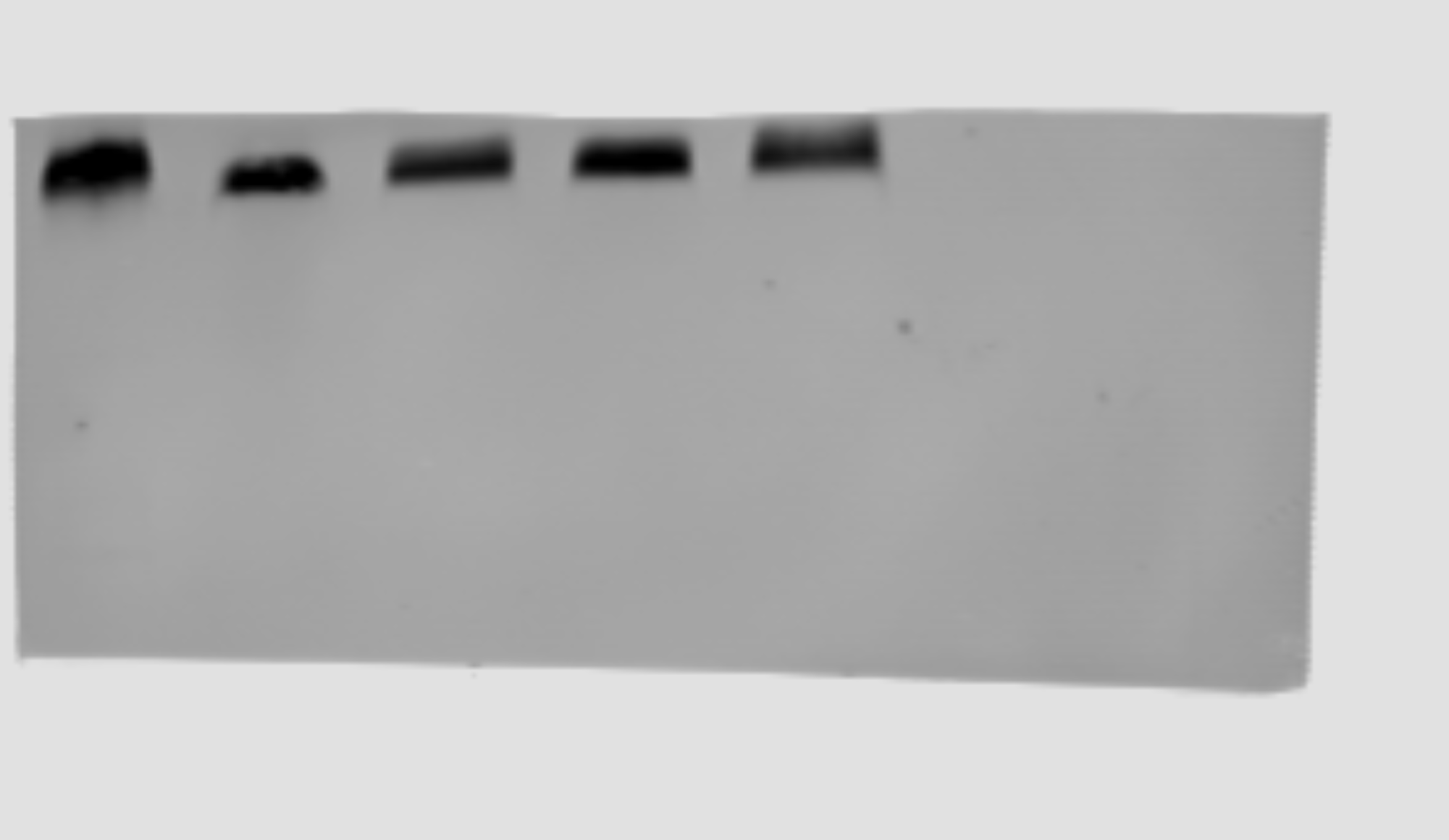

Supplement: Figure 3—source data 1. [file elife-102977-fig3-data1.zip › Figure3-source data 1/Fig3D_HSP90-2_original.tif]

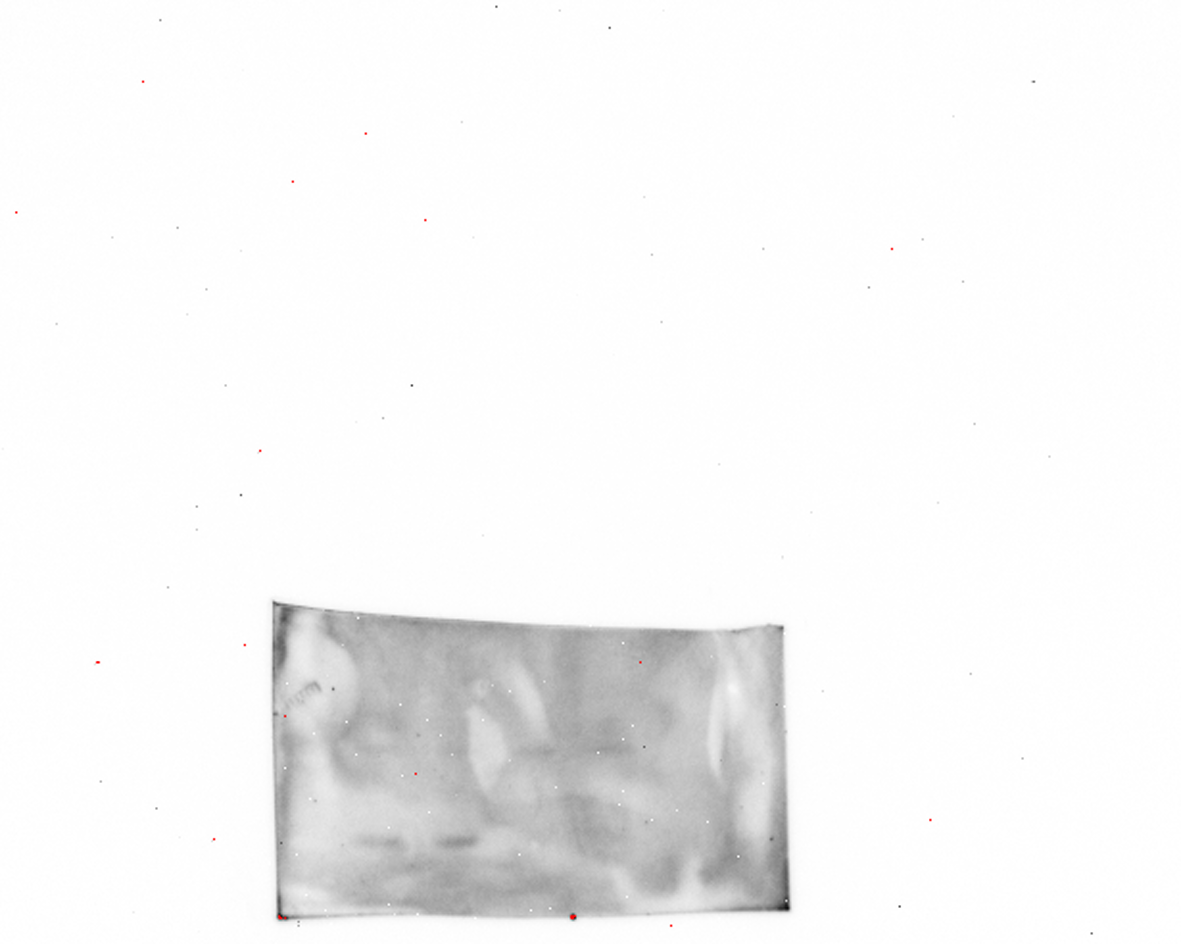

Supplement: Figure 3—source data 1. [file elife-102977-fig3-data1.zip › Figure3-source data 1/Fig3D_ID2_original.tif]

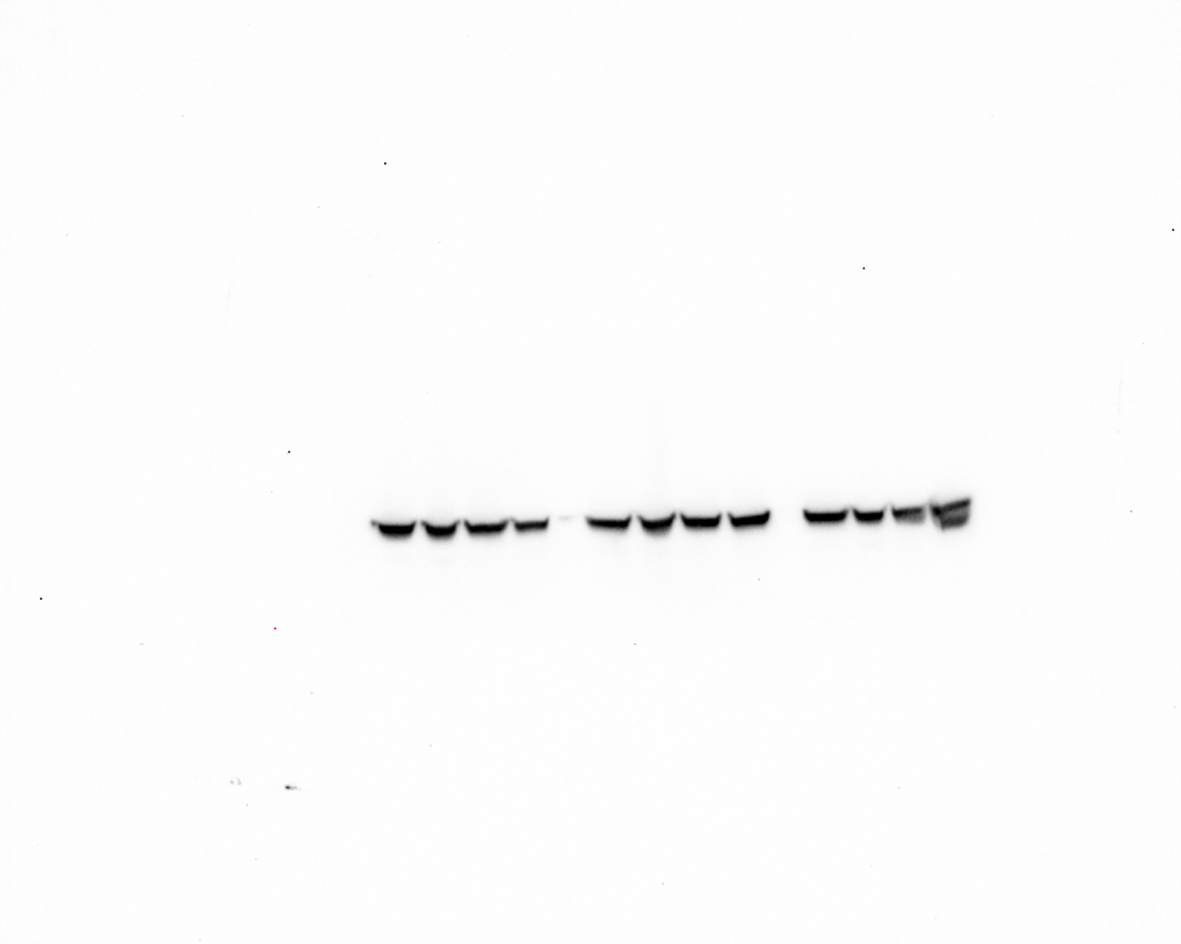

Supplement: Figure 3—source data 1. [file elife-102977-fig3-data1.zip › Figure3-source data 1/Fig3D_HSP90-1_original.tif]

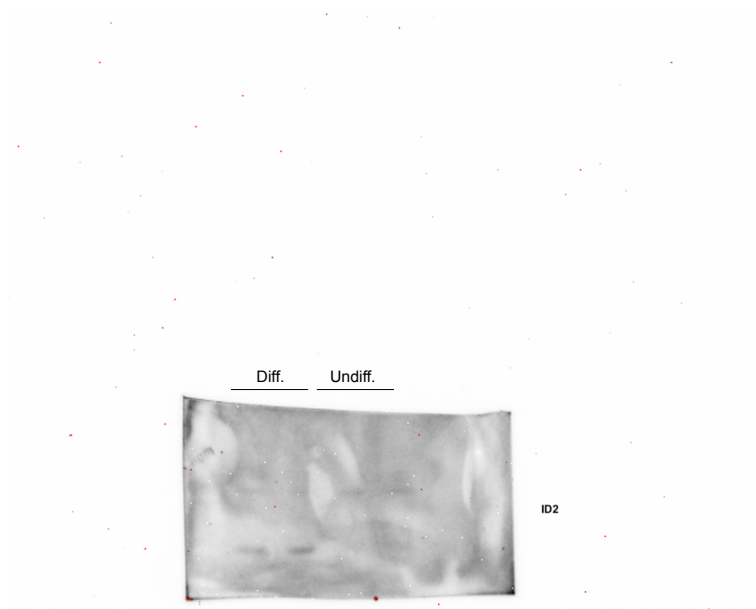

Supplement: Figure 3—source data 2. [file elife-102977-fig3-data2.zip › Figure3-source data 2/Fig3D_ID2_labeled.pdf]

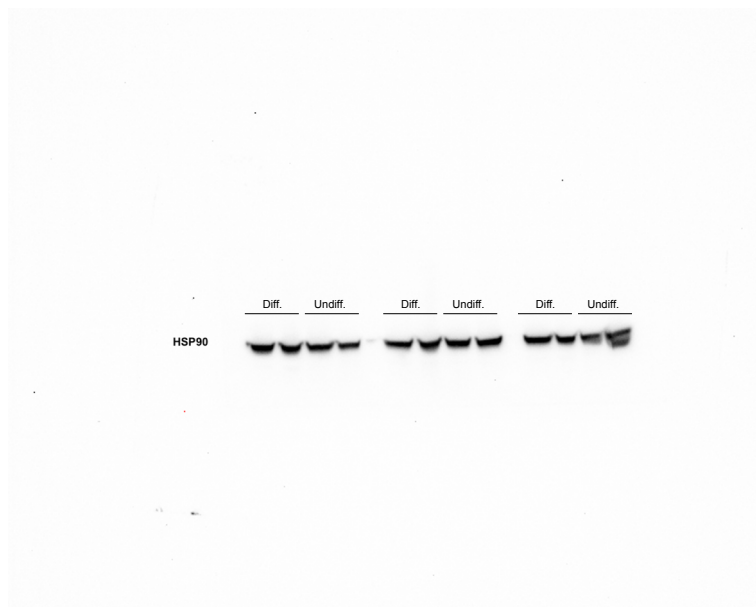

Supplement: Figure 3—source data 2. [file elife-102977-fig3-data2.zip › Figure3-source data 2/Fig3D_HSP90-1_labeled.pdf]

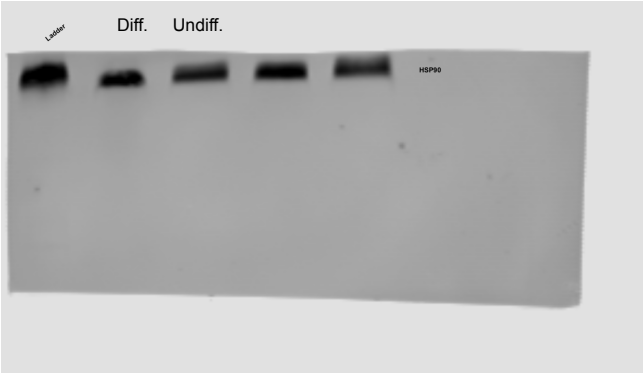

Supplement: Figure 3—source data 2. [file elife-102977-fig3-data2.zip › Figure3-source data 2/Fig3D_HSP90-2_labeled.pdf]

Diff. Undiff.

SLC38A2

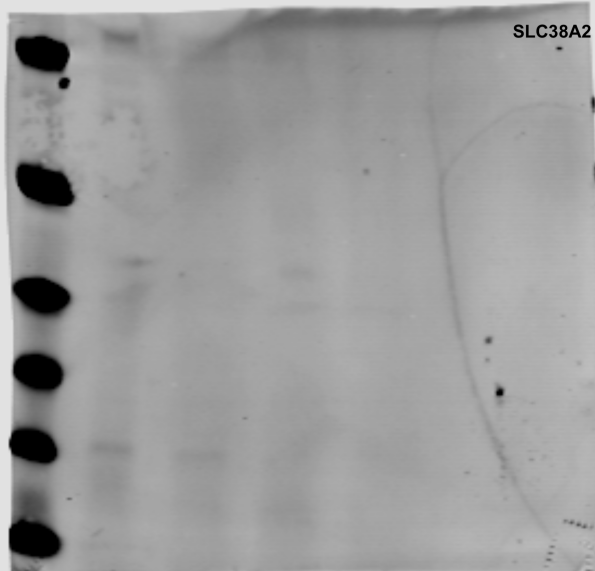

Supplement: Figure 3—source data 2. [file elife-102977-fig3-data2.zip › Figure3-source data 2/Fig3D_SLC38A2_labeled.pdf]

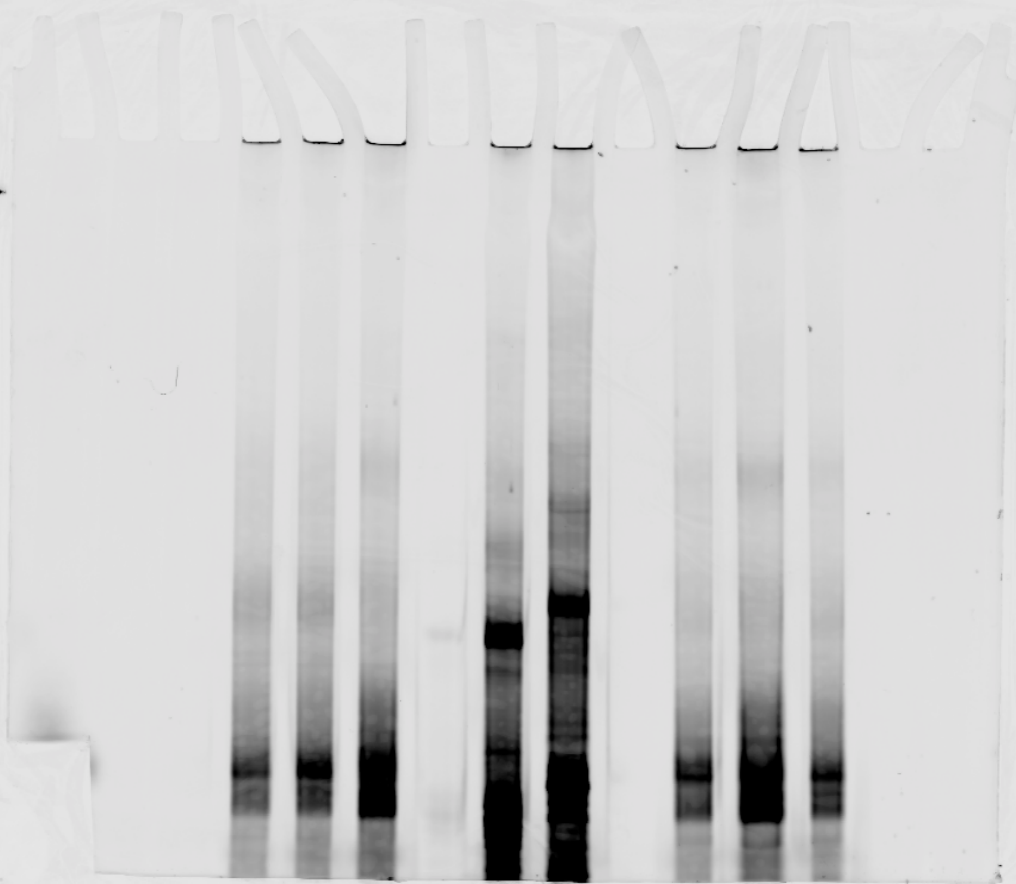

Supplement: Figure 3—figure supplement 1—source data 1. [file elife-102977-fig3-figsupp1-data1.zip › Figure3-figure supplement 1-source data 1/Fig3Supp1_NativeGel_RiboSeq_original.pdf]

Undifferentiated  
NPCs

Ladders

Differentiated  
NPCs

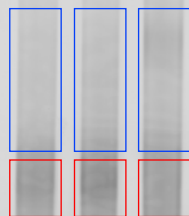

60

30

80

40

Disome Libraries

Monosome Libraries

Supplement: Figure 3—figure supplement 1—source data 2. [file elife-102977-fig3-figsupp1-data2.zip › Figure3-figure supplement 1-source data 2/Fig3Supp1_NativeGel_RiboSeq_labeled.pdf]

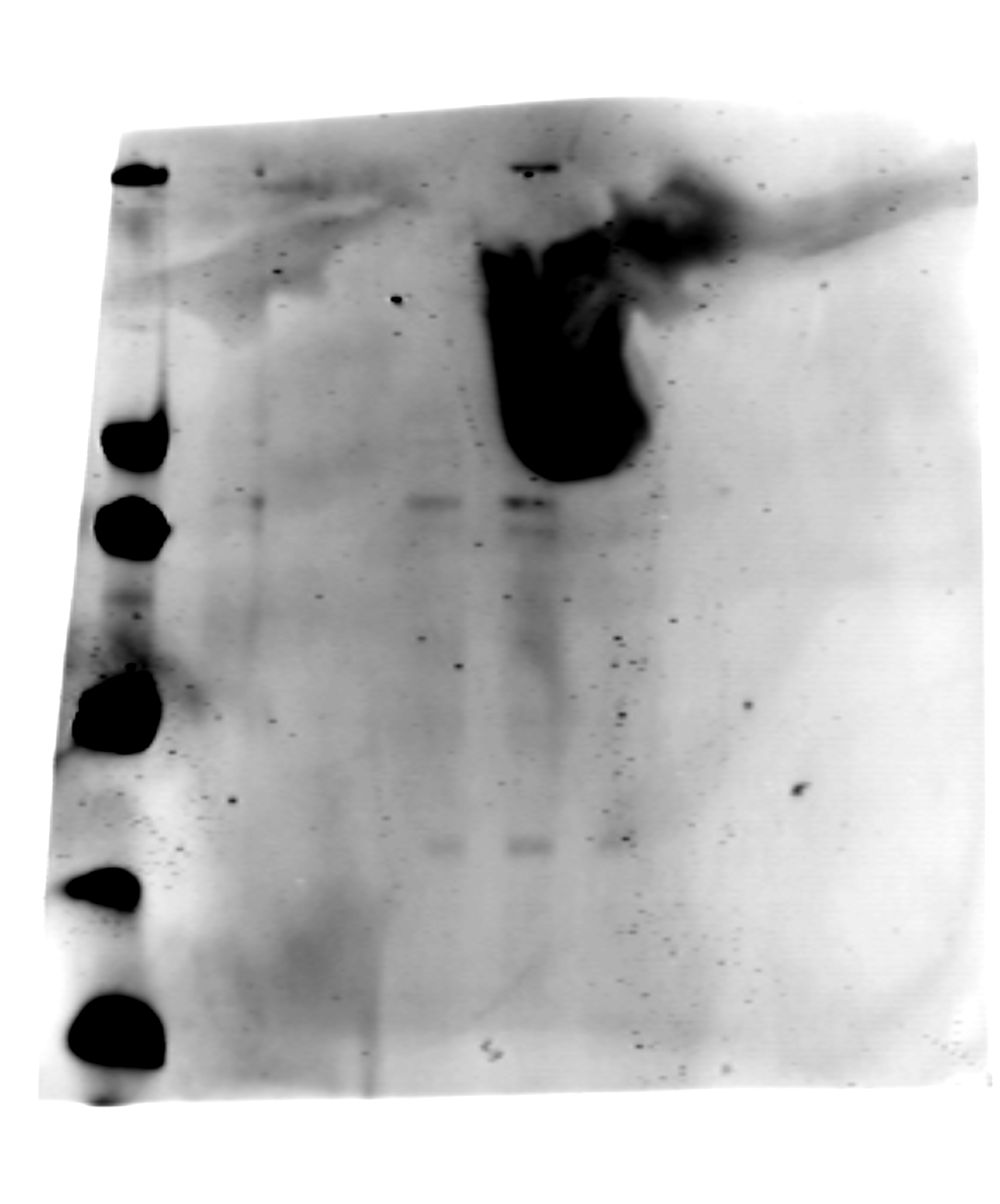

Supplement: Figure 3—figure supplement 3—source data 1. [file elife-102977-fig3-figsupp3-data1.zip › Figure3-figure supplement 3-source data 1/Fig3Supp3_SLC38A2_original.tif]

SLC38A2

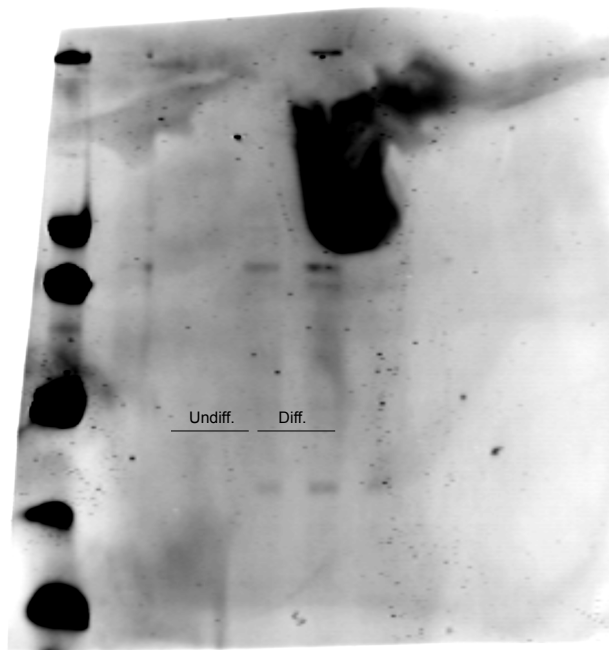

Supplement: Figure 3—figure supplement 3—source data 2. [file elife-102977-fig3-figsupp3-data2.zip › Figure3-figure supplement 3-source data 2/Fig3Supp3_SLC38A2_labeled.pdf]

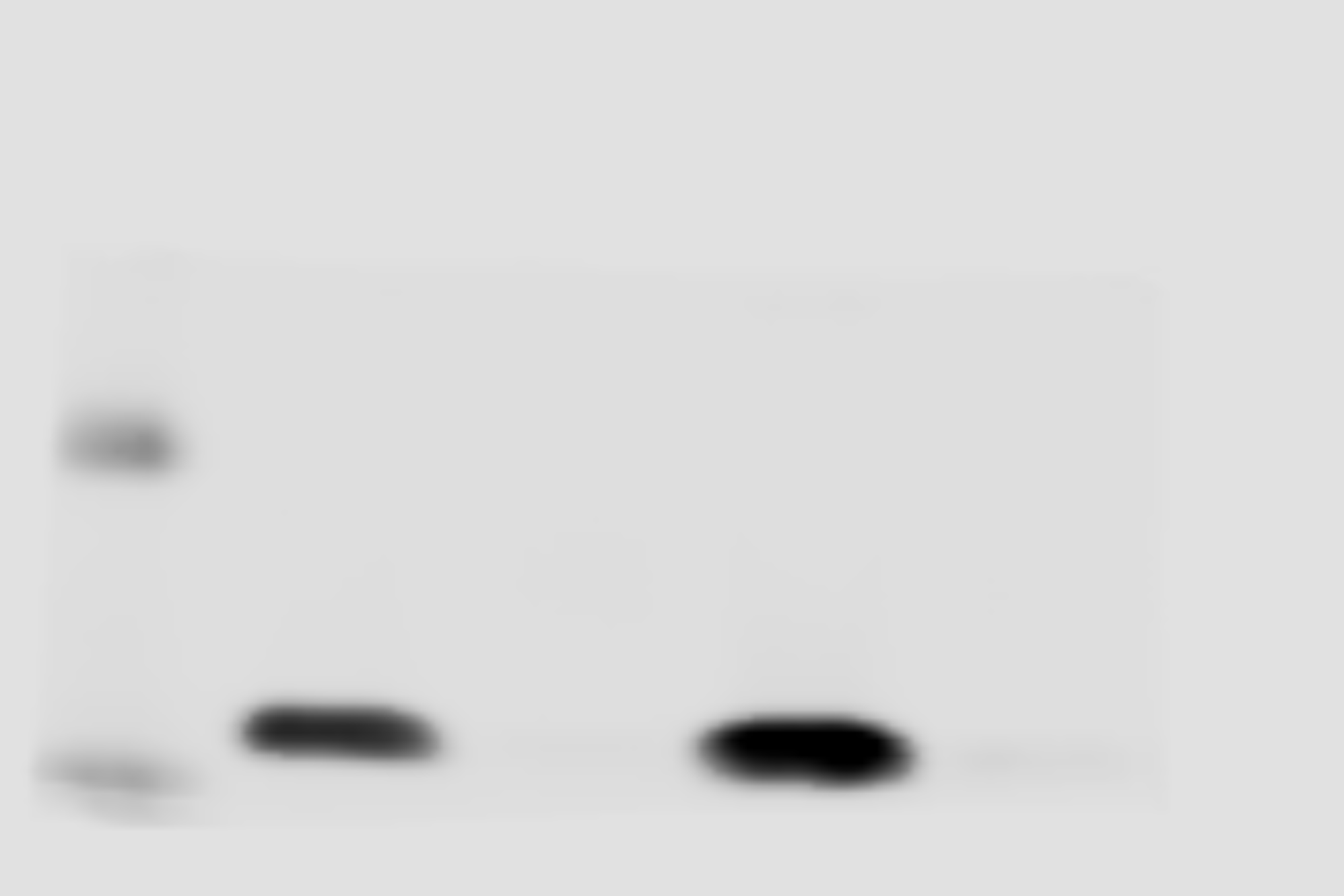

Supplement: Figure 4—source data 1. [file elife-102977-fig4-data1.zip › Figure4-source data 1/FIG4B-Fig4Supp1B_RPS19_original.tif]

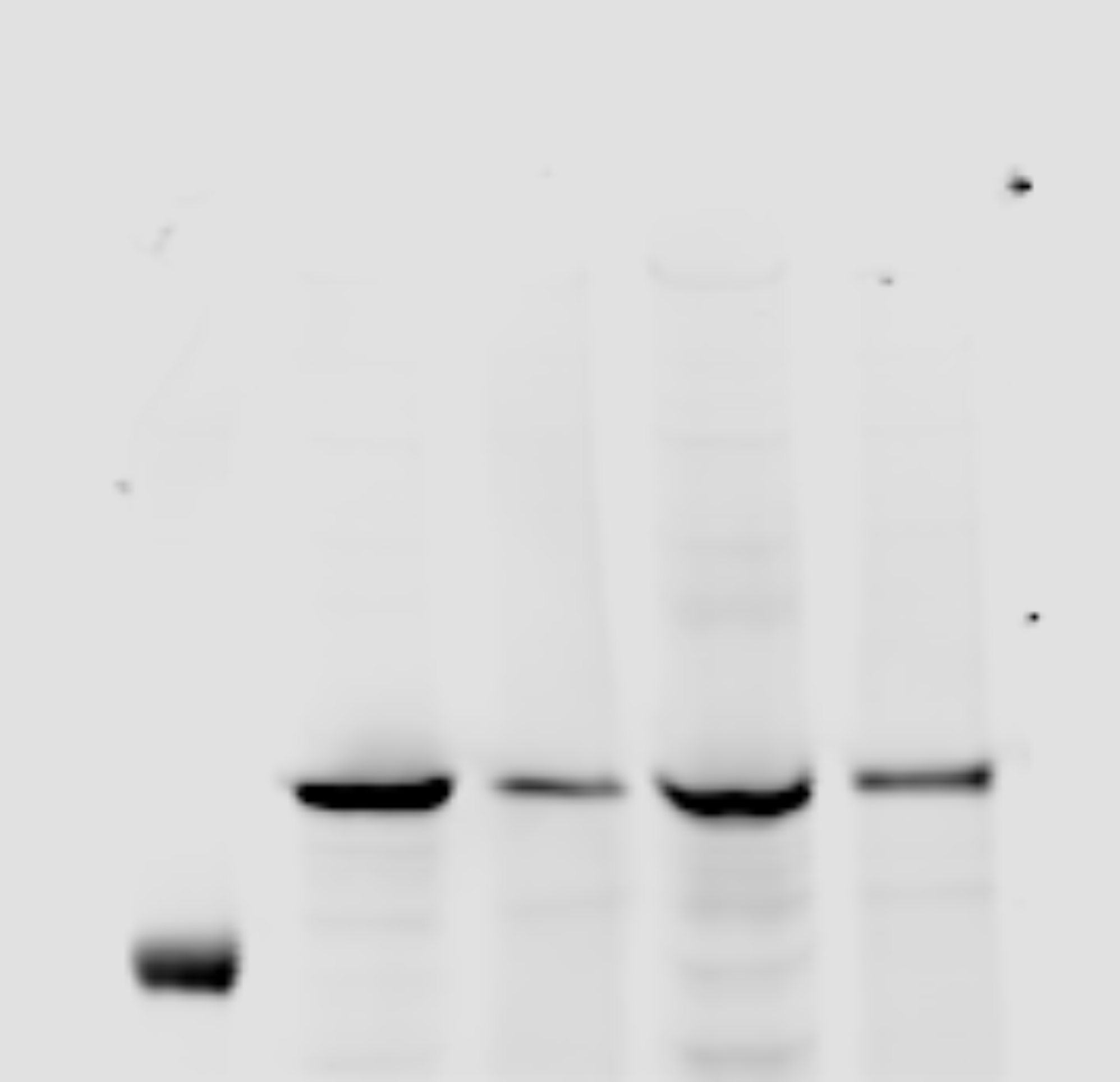

Supplement: Figure 4—source data 1. [file elife-102977-fig4-data1.zip › Figure4-source data 1/Fig4B-Fig4Supp1B_EIF3B_original.tif]

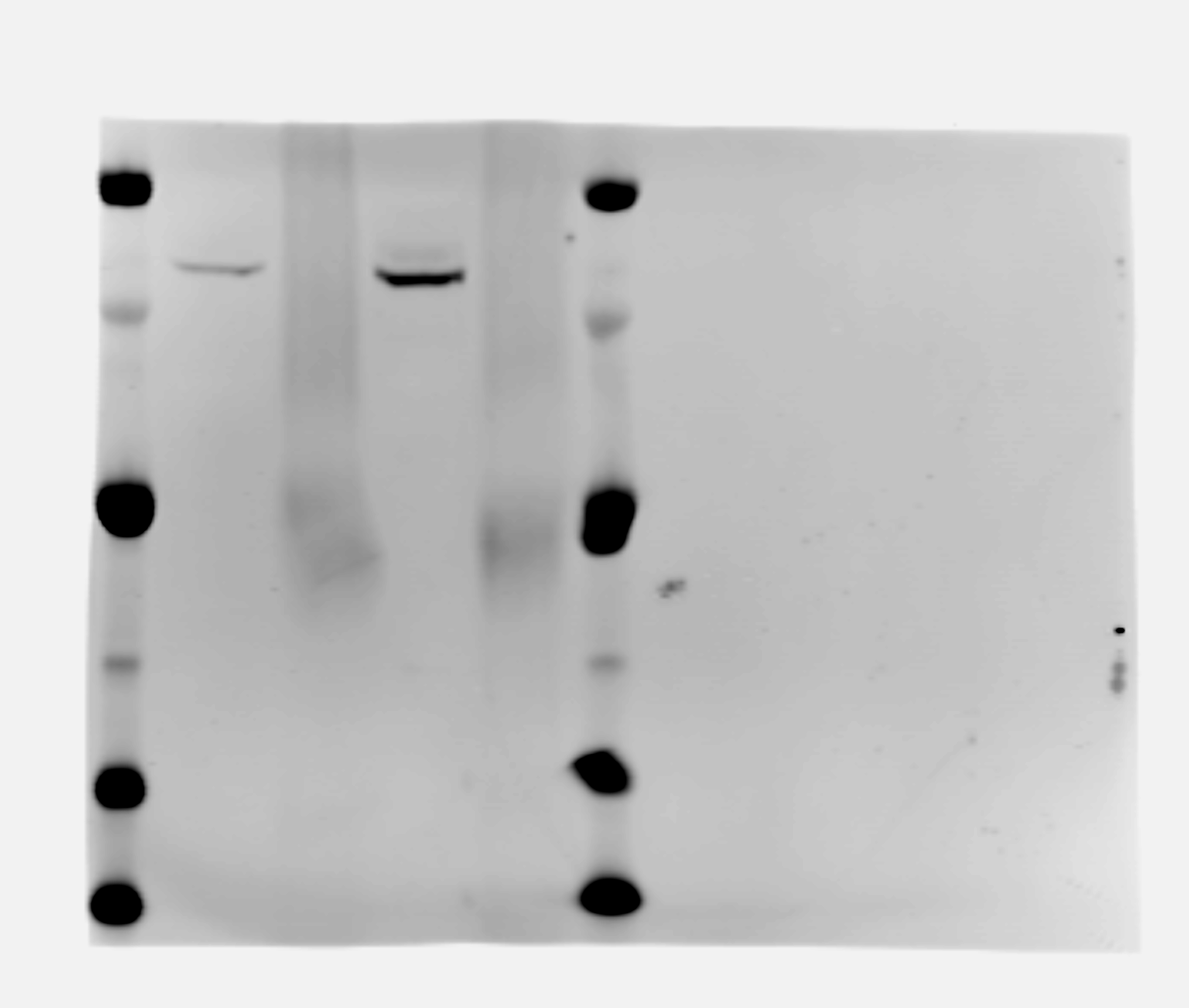

Supplement: Figure 4—source data 1. [file elife-102977-fig4-data1.zip › Figure4-source data 1/Fig4B-Fig4Supp1B_PABPC1_original.tif]

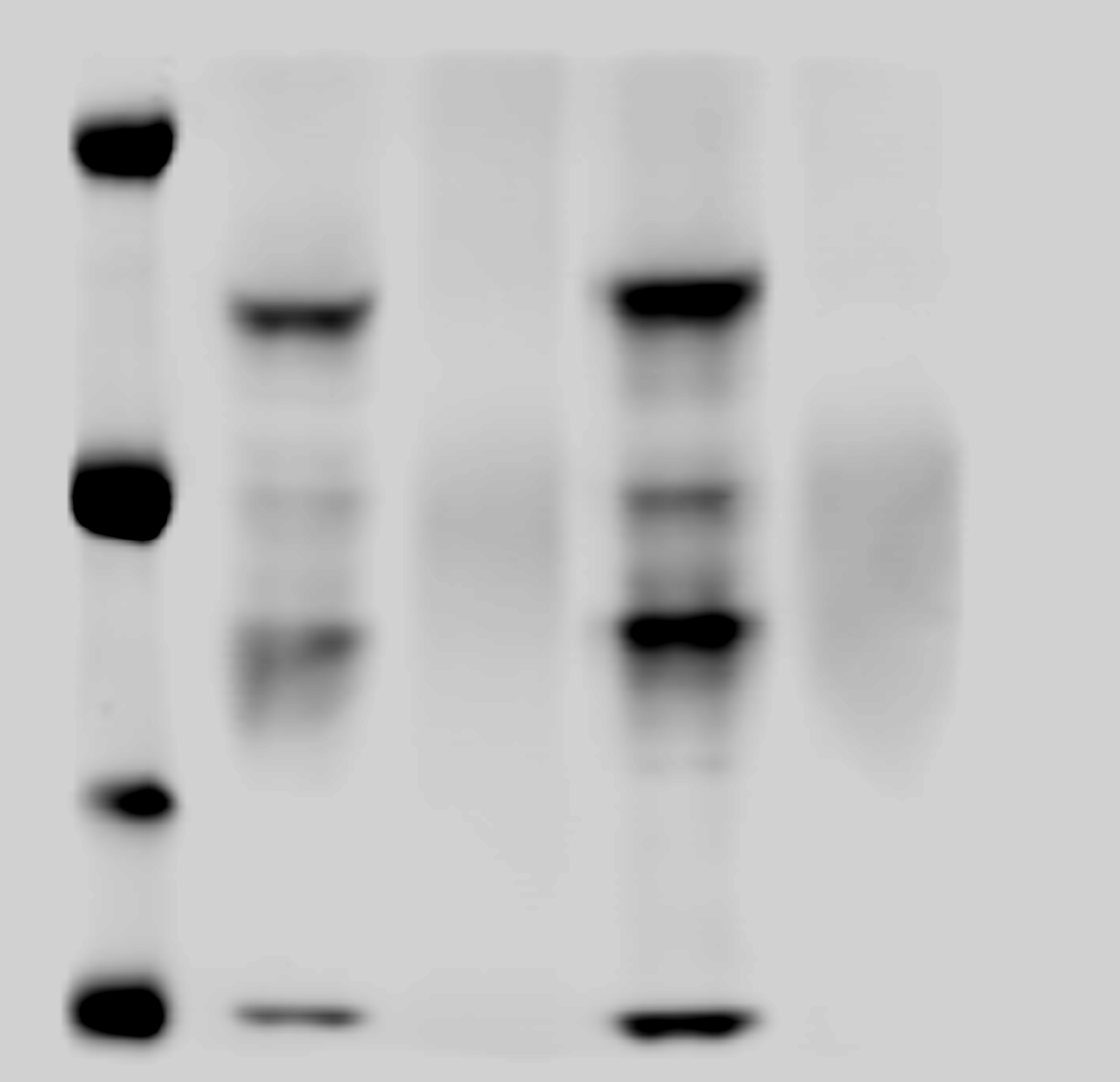

Supplement: Figure 4—source data 1. [file elife-102977-fig4-data1.zip › Figure4-source data 1/Fig4B-Fig4Supp1B_PAIP_original.tif]

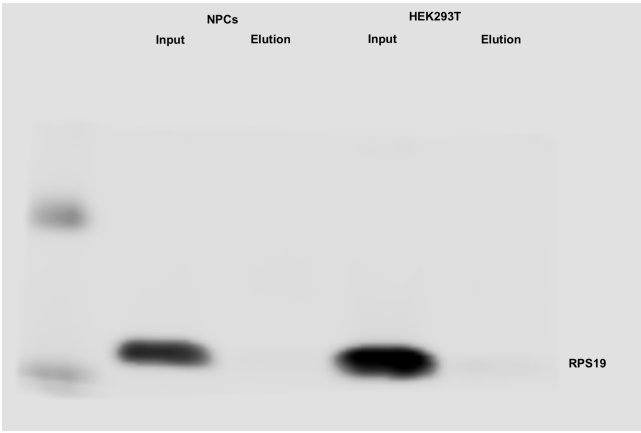

Supplement: Figure 4—source data 2. [file elife-102977-fig4-data2.zip › Figure4-source data 2/Fig4B-Fig4Supp1B_RPS19_labeled.pdf]

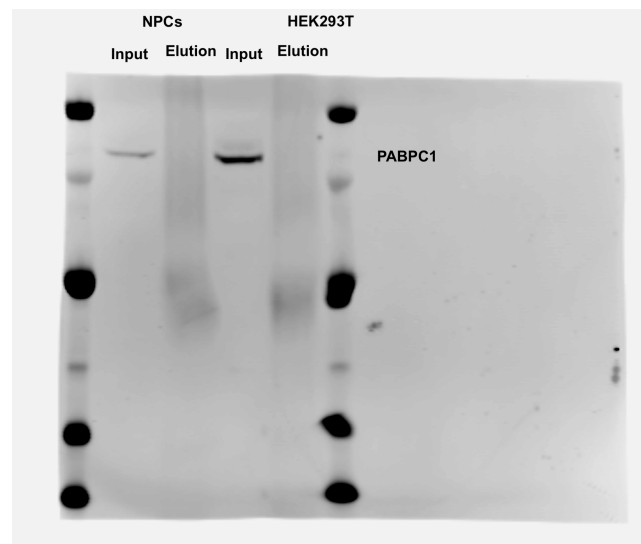

Supplement: Figure 4—source data 2. [file elife-102977-fig4-data2.zip › Figure4-source data 2/Fig4B-Fig4Supp1B_PABPC1_labeled.pdf]

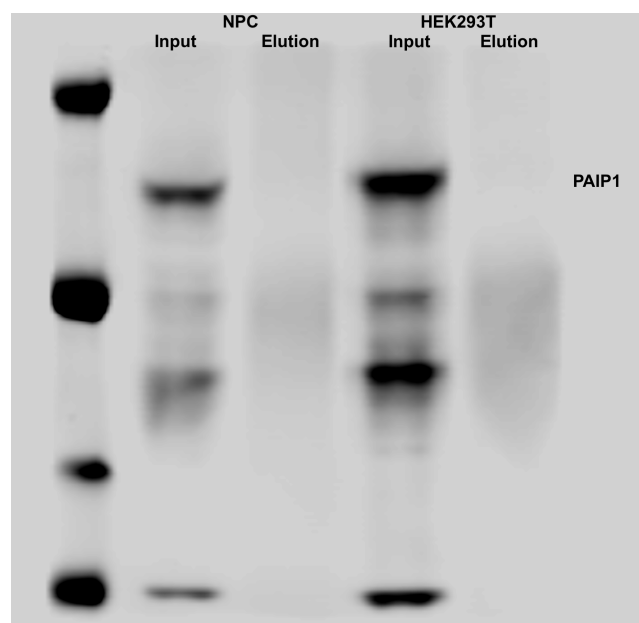

Supplement: Figure 4—source data 2. [file elife-102977-fig4-data2.zip › Figure4-source data 2/Fig4B-Fig4Supp1B_PAIP1_labeled.pdf]

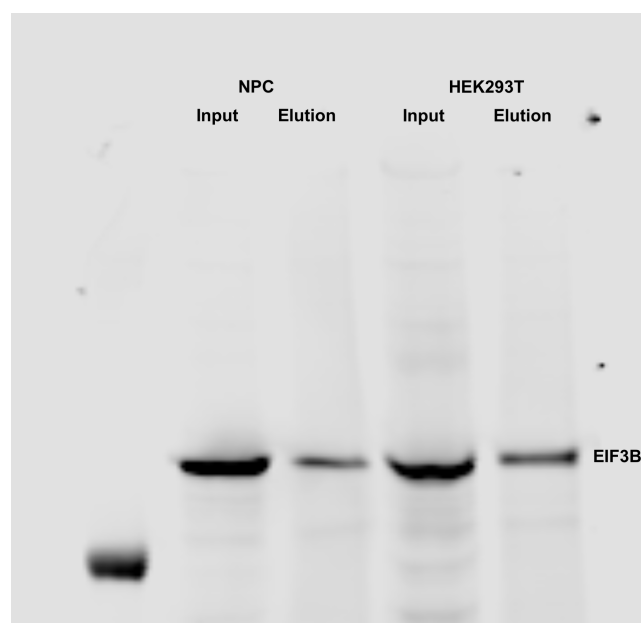

Supplement: Figure 4—source data 2. [file elife-102977-fig4-data2.zip › Figure4-source data 2/Fig4B-Fig4Supp1B_EIF3B_labeled.pdf]

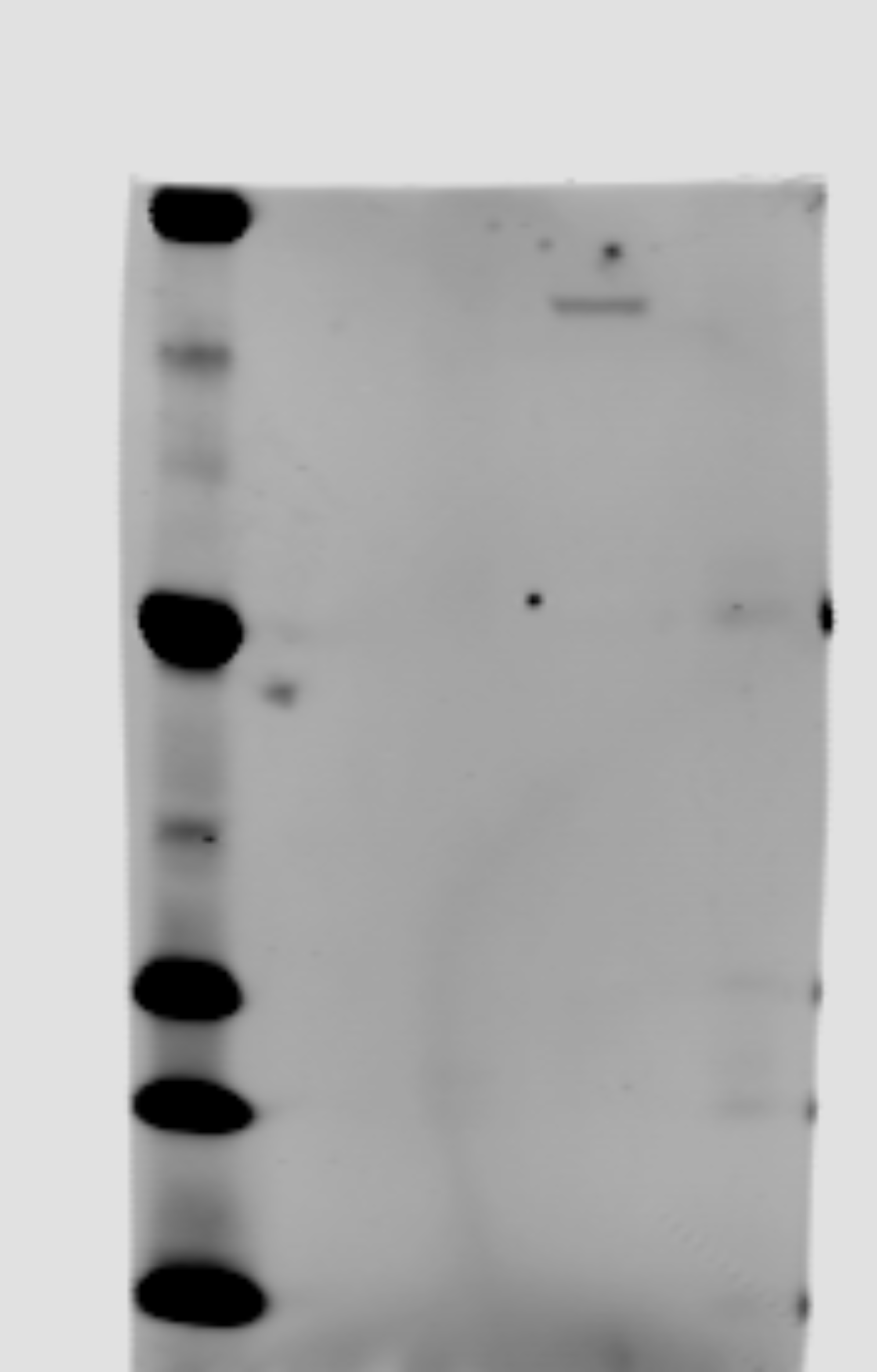

Supplement: Figure 4—figure supplement 1—source data 1. [file elife-102977-fig4-figsupp1-data1.zip › Figure4-figure supplement 1-source data 1/Fig4Supp1C_PABPC1_NPC_HEK293T_original.tif]

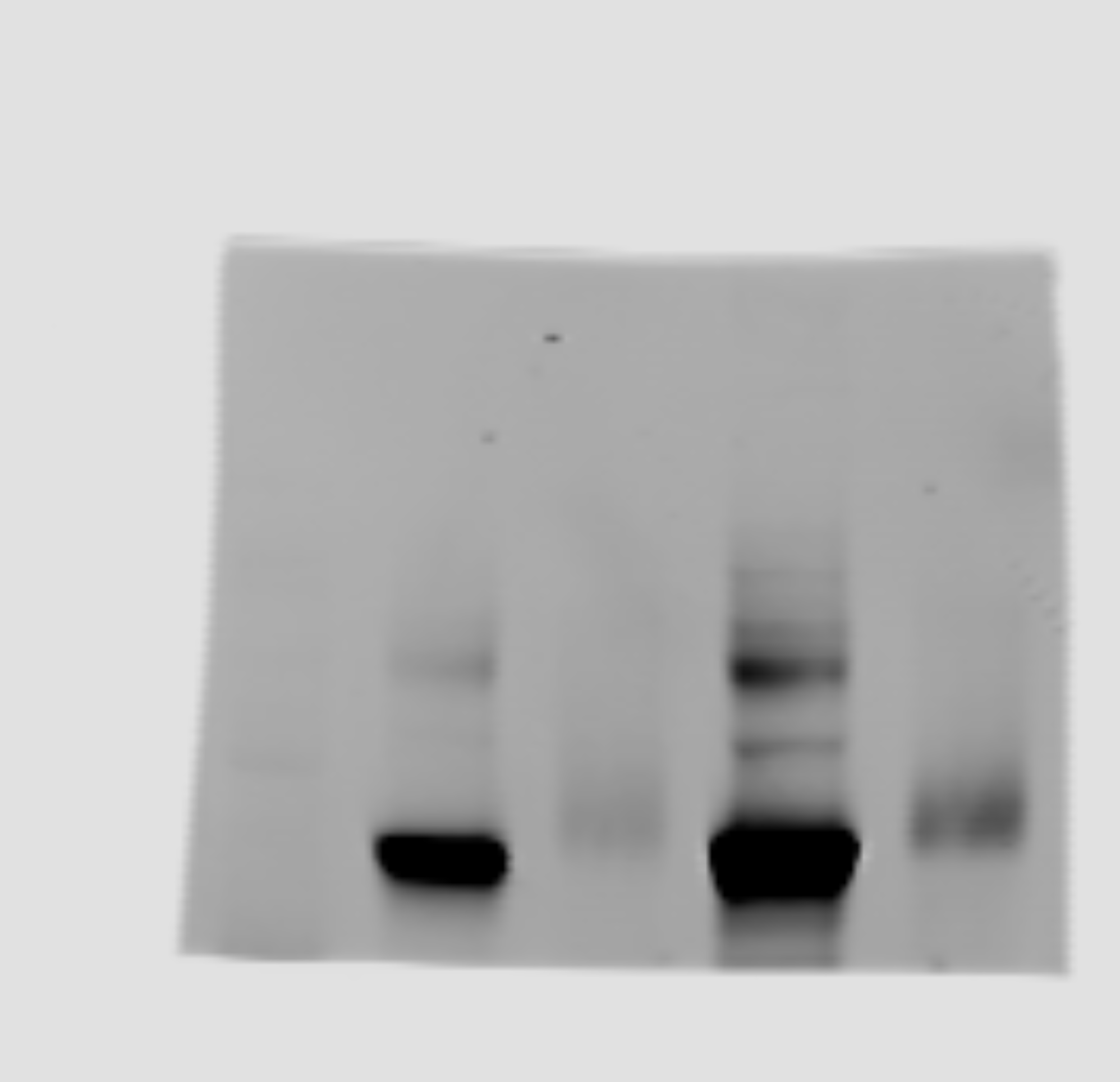

Supplement: Figure 4—figure supplement 1—source data 1. [file elife-102977-fig4-figsupp1-data1.zip › Figure4-figure supplement 1-source data 1/Fig4Supp1C_EIF3B_NPC_HEK293T_original.tif]

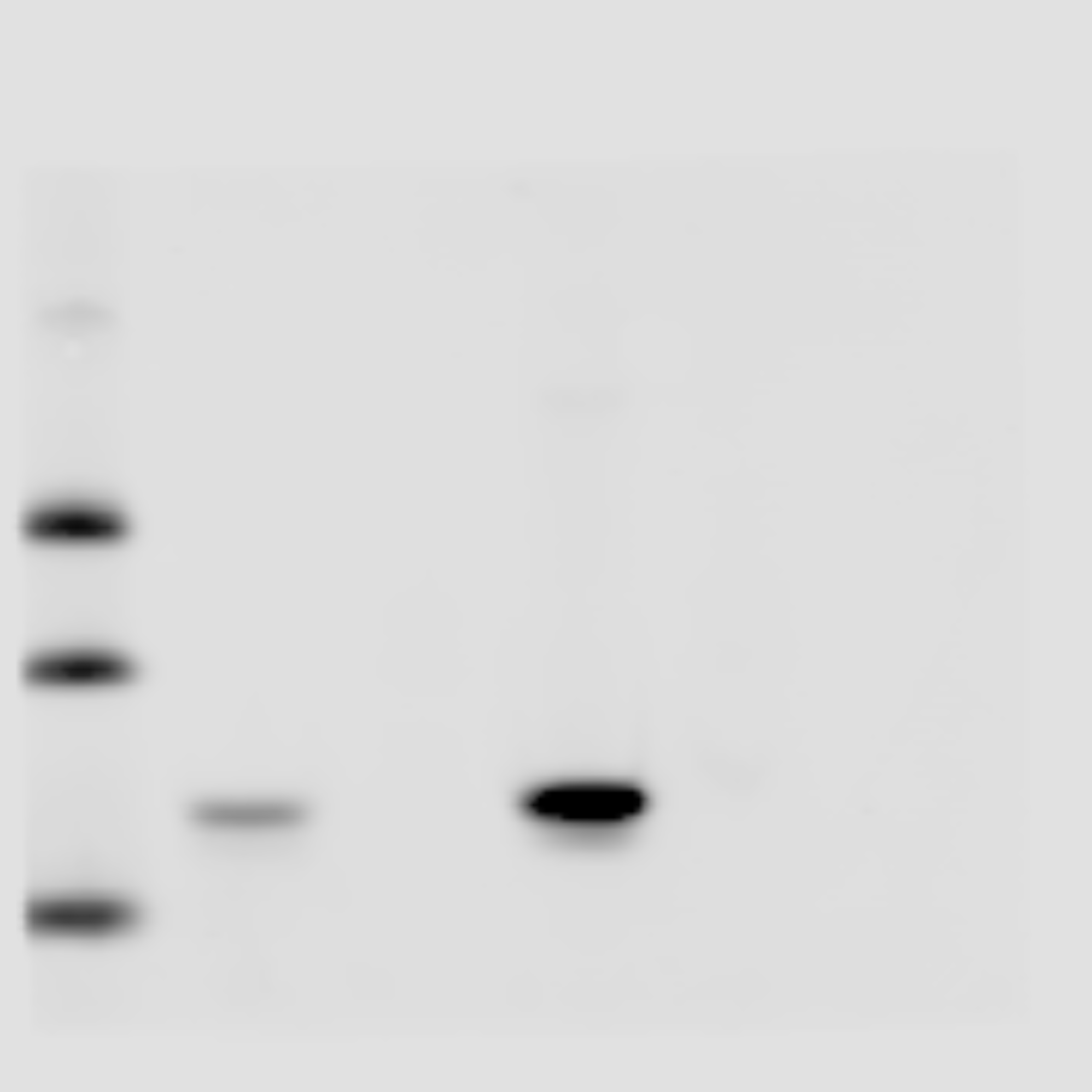

Supplement: Figure 4—figure supplement 1—source data 1. [file elife-102977-fig4-figsupp1-data1.zip › Figure4-figure supplement 1-source data 1/Fig4Supp1C_RPS19_NPC_HEK293T_original.tif]

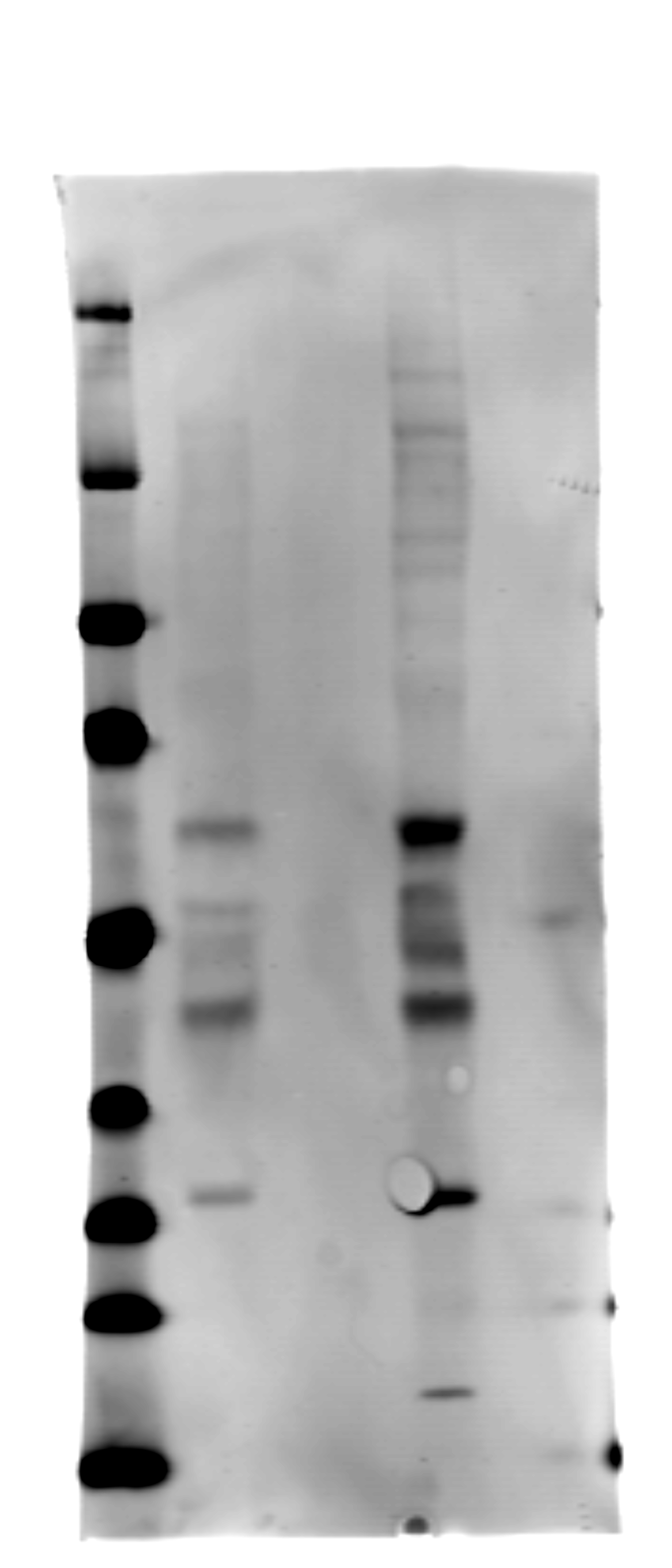

Supplement: Figure 4—figure supplement 1—source data 1. [file elife-102977-fig4-figsupp1-data1.zip › Figure4-figure supplement 1-source data 1/Fig4Supp1C_PAIP1_NPC_HEK293T_original.tif]

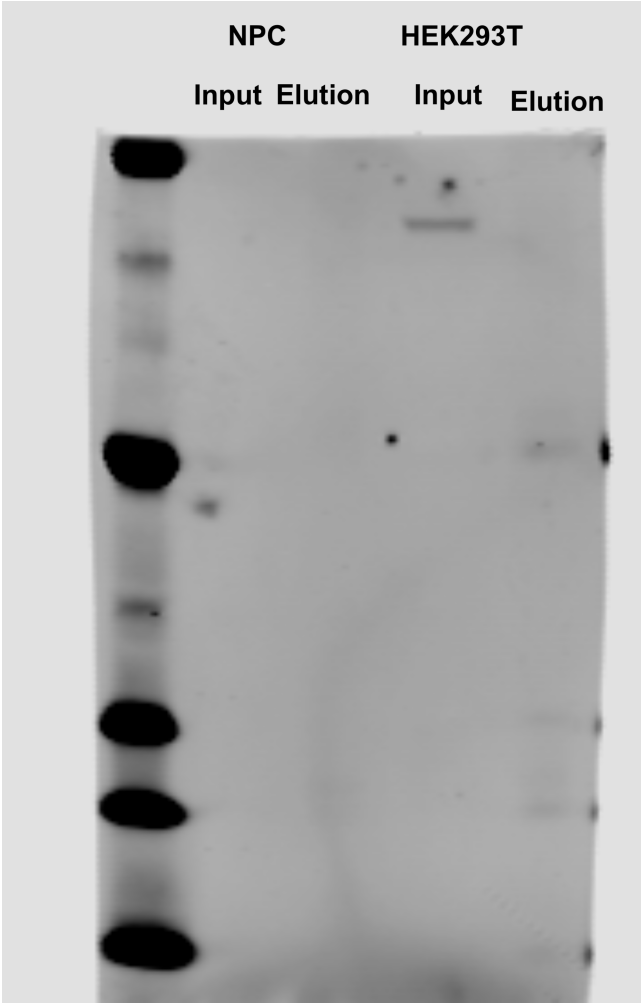

Supplement: Figure 4—figure supplement 1—source data 2. [file elife-102977-fig4-figsupp1-data2.zip › Figure4-figure supplement 1-source data 2/Fig4Supp1C_PABPC1_NPC_HEK293T_labeled.pdf]

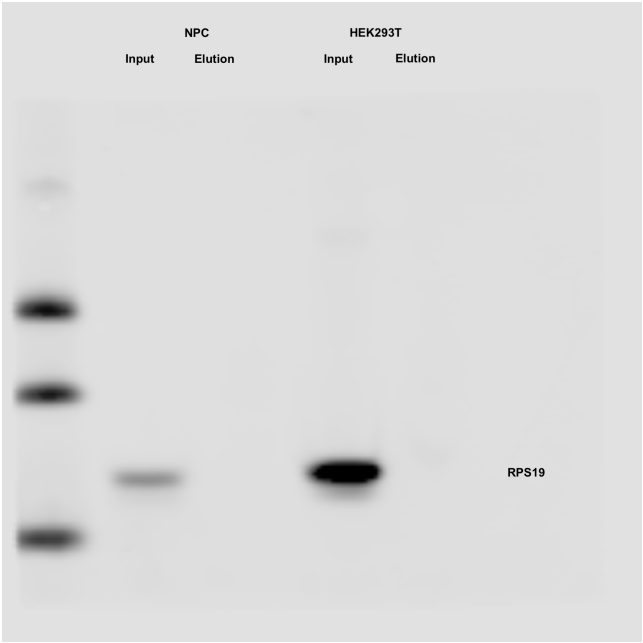

Supplement: Figure 4—figure supplement 1—source data 2. [file elife-102977-fig4-figsupp1-data2.zip › Figure4-figure supplement 1-source data 2/Fig4Supp1C_RPS19_NPC_HEK293T_labeled.pdf]

NPC

HEK293T

Input Elution Input Elution

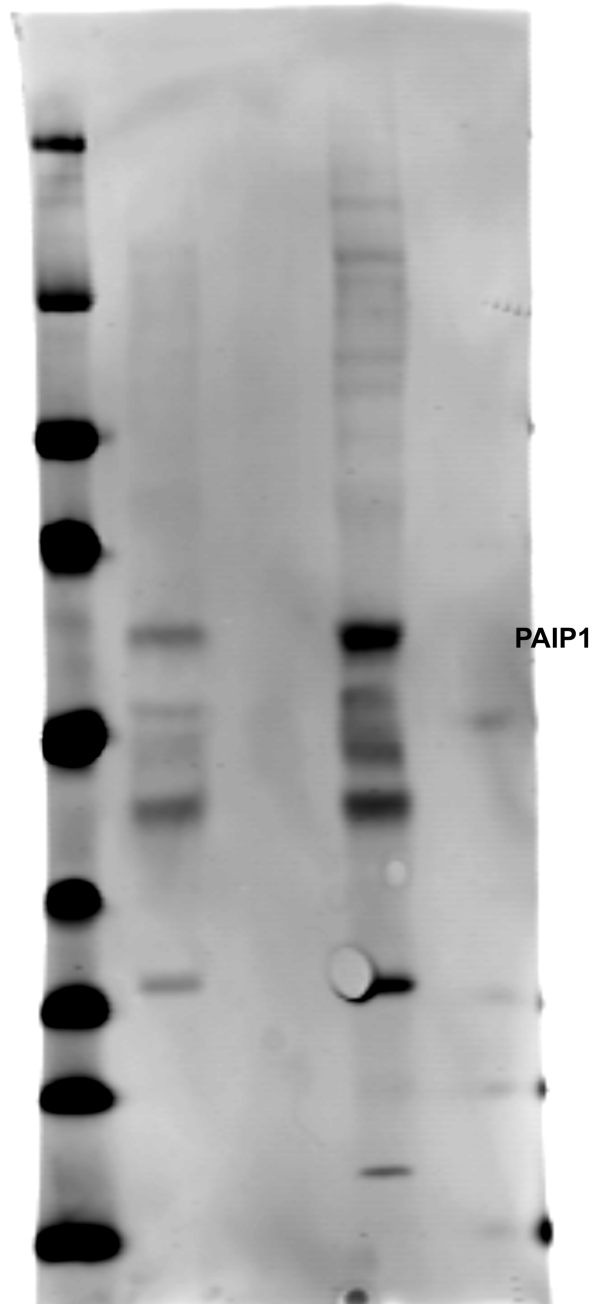

Supplement: Figure 4—figure supplement 1—source data 2. [file elife-102977-fig4-figsupp1-data2.zip › Figure4-figure supplement 1-source data 2/Fig4Supp1C_PAIP1_NPC_HEK293T_labeled.pdf]

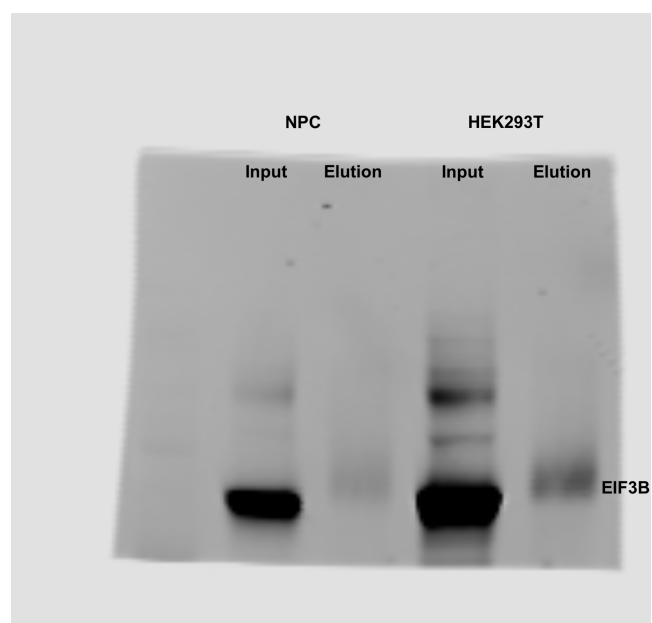

Supplement: Figure 4—figure supplement 1—source data 2. [file elife-102977-fig4-figsupp1-data2.zip › Figure4-figure supplement 1-source data 2/Fig4Supp1C_EIF3B_NPC_HEK293T_labeled.pdf]
