# Supplementary material for: eIF3 engages with 3’-UTR termini of highly translated mRNAs
Source: eLife. 2025 Jan 29;13:RP102977. doi: 10.7554/eLife.102977 (PMC11778930; doi:10.7554/eLife.102977)
Supplement: Figure 1—figure supplement 1—source data 2. [file elife-102977-fig1-figsupp1-data2.zip › Figure1-figure supplement 1-source data 2/Fig1Supp1B_puromycin_totalproteinstain_labeled.pdf]

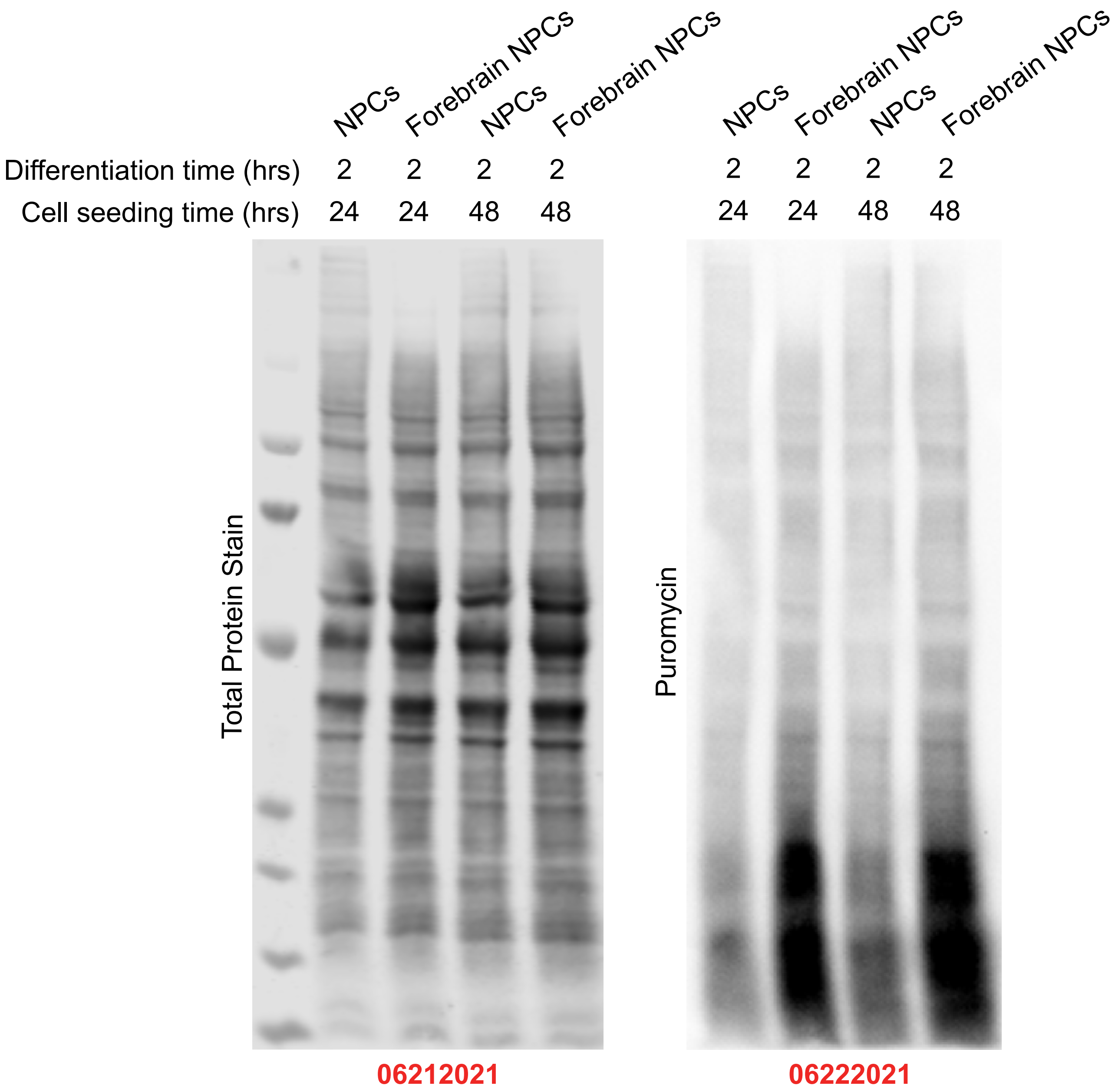
NPCs
Forebrain NPCs
NPCs
Forebrain NPCs
NPCs
Forebrain NPCs
NPCs
Forebrain NPCs
Differentiation time (hrs) 2 2 2 2 2 2 2 2
Cell seeding time (hrs) 24 24 48 48 24 24 48 48
Total Protein Stain
Puromycin
06212021
06222021
