# Supplementary material for: eIF3 engages with 3’-UTR termini of highly translated mRNAs
Source: eLife. 2025 Jan 29;13:RP102977. doi: 10.7554/eLife.102977 (PMC11778930; doi:10.7554/eLife.102977)
Supplement: Figure 1—figure supplement 5—source data 2. [file elife-102977-fig1-figsupp5-data2.zip › Figure1-figure supplement 5-source data 2/Fig1Supp5_IR-RNAs_RPS19_labeled.pdf]

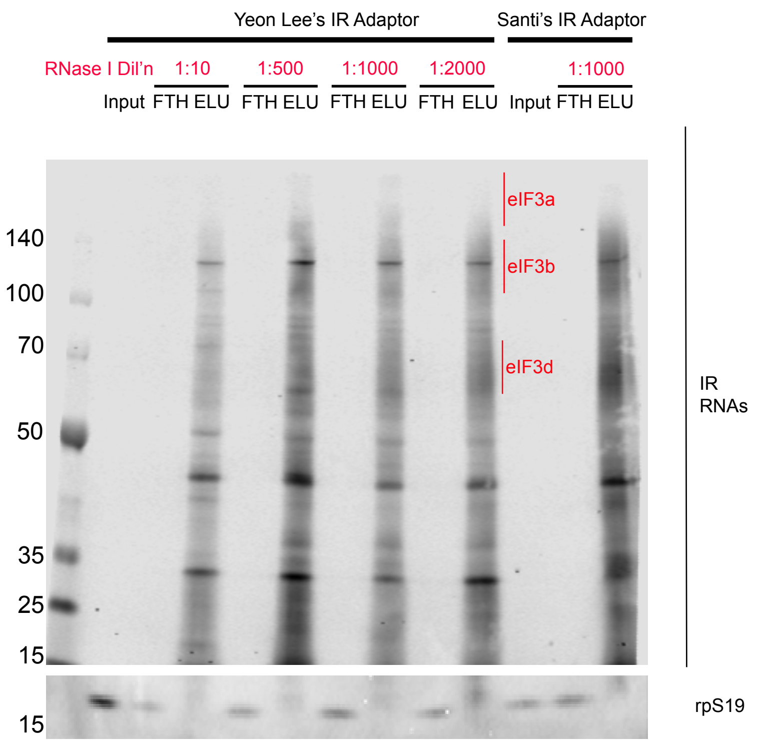

Yeon Lee's IR Adaptor
Santi's IR Adaptor
RNase I Dil'n
1:10
1:500
1:1000
1:2000
1:1000
Input
FTH ELU
FTH ELU
FTH ELU
FTH ELU
Input
FTH ELU
140
100
70
50
35
25
15
eIF3a
eIF3b
eIF3d
IR RNAs
15
rpS19
